# Genome Analysis Reveals Genetic Admixture and Signature of Selection for Productivity and Environmental Traits in Iraqi Cattle

**DOI:** 10.3389/fgene.2019.00609

**Published:** 2019-07-16

**Authors:** Akil Alshawi, Abdulameer Essa, Sahar Al-Bayatti, Olivier Hanotte

**Affiliations:** ^1^Division of Cells, Organisms and Molecular Genetics, School of Life Sciences, Faculty of Medicine and Health Sciences, University Park Campus, University of Nottingham, Nottingham, United Kingdom; ^2^Department of Internal and Preventive Veterinary Medicine, College of Veterinary Medicine, University of Baghdad, Iraqi Ministry of Higher Education and Scientific Research, Baghdad, Iraq; ^3^Animal Genetics Resources Department, Directorate of Animal Resources, the Ministry of Iraqi Agriculture, Baghdad, Iraq; ^4^LiveGene, International Livestock Research Institute (ILRI), Addis Ababa, Ethiopia

**Keywords:** *Bos taurus*, *Bos indicus*, genetic structure, diversity, positive selection, immune responses, adaptive genes

## Abstract

The Near East cattle are adapted to different agro-ecological zones including desert areas, mountains habitats, and humid regions along the Tigris and Euphrates rivers system. The region was one of the earliest and most significant areas of cattle husbandry. Currently, four main breeds of Iraqi cattle are recognized. Among these, the Jenoubi is found in the southern more humid part of Iraq, while the Rustaqi is found in the middle and drier region of the country. Despite their importance, Iraqi cattle have up to now been poorly characterized at the genome level. Here, we report at a genome-wide level the diversity and signature of positive selection in these two breeds. Thirty-five unrelated Jenoubi cattle, sampled in the Maysan and Basra regions, and 60 Rustaqi cattle, from around Baghdad and Babylon, were genotyped using the Illumina Bovine HD BeadChip (700K). Genetic population structure and diversity level were studied using principal component analysis (PCA), expected heterozygosity (*He*), observed heterozygosity (*Ho*), and admixture. Signatures of selection were studied using extended haplotype homozygosity (EHH) (*iHS* and *Rsb*) and inter-population Wright’s *Fst*. The results of PCA and admixture analysis, including European taurine, Asian indicine, African indicine, and taurine indicate that the two breeds are crossbreed zebu × taurine, with more zebu background in Jenoubi cattle compared with Rustaqi. The Rustaqi has the greatest mean heterozygosity (*He* = 0.37) among all breeds. *iHS* and *Rsb* signatures of selection analyses identify 68 candidate genes under positive selection in the two Iraqi breeds, while *Fst* analysis identifies 220 candidate genes including genes related to the innate and acquired immunity responses, different environmental selection pressures (e.g., tick resistance and heat stress), and genes of commercial interest (e.g., marbling score).

## Introduction

Archeological and genetic studies support two centers of cattle domestication, the Fertile Crescent and the Northern part of the Indian subcontinent including the Indus Valley (Loftus et al., 1994; Bradley et al., 1998; Troy et al., 2001; Helmer et al., 2005; Bradley and Magee, 2006; Zeder, 2008; Chen et al., 2010; Magee et al., 2014). The earliest archeological evidence of humpless cattle was found in the Fertile Crescent, dated to around 10,000 bc. The first evidence of domestic humped cattle is from the Indus Valley region around 8,000 bc (Felius et al., 2015). From these two heartlands of domestication, two main cattle types, *Bos taurus* (humpless taurine) and *Bos indicus* (humped zebu), dispersed across the world, with taurine cattle reaching Africa, Europe, and East Asia and indicine cattle migrating to Africa, South Asia, and South-East Asia (Hanotte et al., 2002; Diamond and Bellwood, 2003; Freeman et al., 2006a; Gifford-Gonzalez and Hanotte, 2011; Magee et al., 2014). The domestication process of animals was essentially a form of symbiosis with humans enabling the dissemination of domesticated cattle throughout the world (Diamond and Bellwood, 2003).

Cattle husbandry was part of the ancient civilizations of Mesopotamia, modern-day Iraq, at an early time with the earliest available evidence of domestic cattle in this region dating to around 6000 bc. There are many archeological evidence of the antiquity and importance of cattle husbandry in central Mesopotamia, including cylinder seals (Read, 2015). These animals were of the humpless taurine type. Further south, closer to the Indus Valley center of cattle domestication, archeological evidence of domestic cattle in Mesopotamia is much fewer. It includes artistic depictions from the royal tombs of Ur (South of Iraq) including domestic animals (**Supplementary Figure S1A, B**), with most of the ancient agricultural settlements along the Tigris and Euphrates now buried under flooded plains (Read, 2015).

Despite the high cattle number worldwide, it is estimated that 17% of cattle breeds are facing extinction, following changing environmental and production conditions (Rischkowsky and Pilling, 2007; Taberlet et al., 2011; FAO, 2015; Felius et al., 2015). Endangered breeds of cattle are mostly found in developing countries (Joost et al., 2015). For instance, 32% of the recognized indigenous African breeds are at risk of extinction, and another 22% are already considered extinct (Mwai et al., 2015).

Several studies have explored the genome diversity and adaptation of cattle breeds using either high-density SNP chips (Bovine high density SNPs BeadChip (777,962 SNPs) (e.g., Xu et al., 2014; Bahbahani et al., 2017) or full genome sequence analysis (e.g., Choi et al., 2015; Kim et al., 2017). However, the cattle genomes of the Fertile Crescent (including Iraq) have not yet been characterized. The available studies are a microsatellite study (Ates¸ et al., 2014) and mitochondrial DNA analysis for a few breeds (Freeman et al., 2006a, Freeman et al., 2006b; Edwards et al., 2007; Ateş et al., 2014). These studies show evidence of zebu introgression within the Near East taurine, in particular within the Iraqi and Anatolian breeds.

Today, four cattle breeds are officially recognized in Iraq (Al-Murrani et al., 2003) (**Supplementary Table S1**). Karradi and Sharabi in the northern part of the country, Rustaqi in the central part, and further south, Jenoubi. Phenotypically, Rustaqi may be classified as taurine and Jenoubi as zebu. Living in different agro-ecologies, they will be expected to be adapted to different environmental challenges, including external–internal parasites or infectious diseases, heat, and humidity. Alongside, some commercial breeds have been introduced over the years, including Jersey, Hereford, Ayrshire, and Holstein-Friesian cattle (Al-Murrani et al., 2003) with crossbreeding between Sharabi and Friesian cattle documented (Dabdoub, 2005; Maaroof, 2011; Nasser et al., 2013; Nassar et al., 2014). Until now, none of these Iraqi native breeds have been documented at a genome-wide level, despite the uniqueness of the country in the region of taurine cattle domestication, and its historic importance as major center of civilization in the past. We report for the first time at the autosomal genome-wide level, the genome diversity and candidate signatures of positive selection (e.g., tick resistance genes) in two Iraqi cattle breeds (Jenoubi and Rustaqi), providing new insights on the past and present breeding dynamics and evolutionary forces that shaped the genome of the cattle population in the region.

## Materials and Methods

### Population Samples

**Figure 1:**
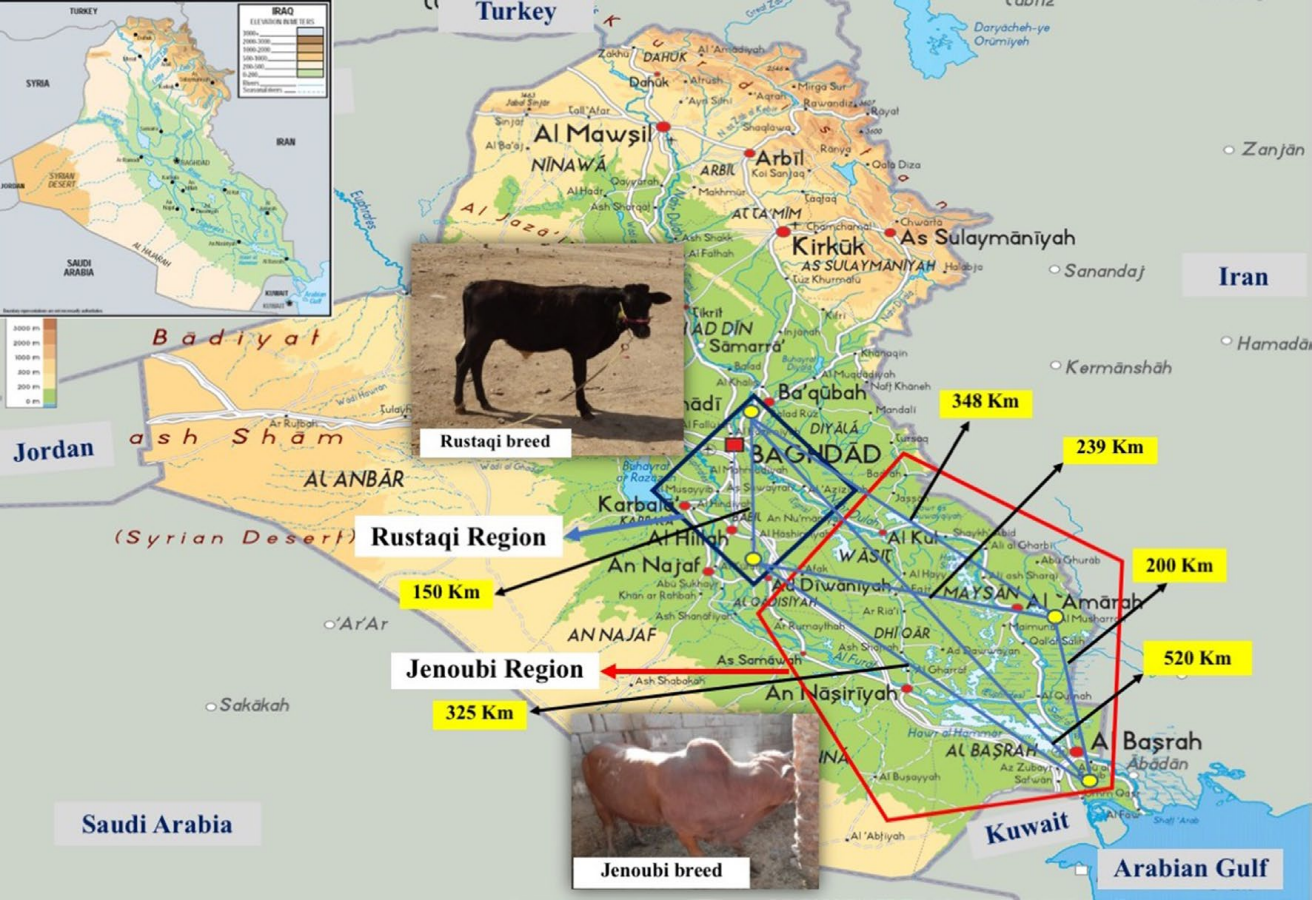
Geographic distribution and sampling location of Rustaqi and Jenoubi breeds. Blue square: Rustaqi breed distribution. Red pentagon: Jenoubi breed distribution. Yellow dots: sampling locations. Geographical distances between sampling locations are indicated in black digits. Physical map obtained from http://www.mapsland.com/asia/iraq.

We collected blood samples spotted on FTA paper (Whatman Technology^®^) from two indigenous Iraqi breeds, Rustaqi and Jenoubi (**Figure 1**). In particular, for the Rustaqi breed, 60 blood samples were collected: 20 samples from the Baghdad region, in central Iraq, and 40 samples from the Babylon area (80 km south of Baghdad). For the Jenoubi breed, 35 blood samples were collected from the southern regions of Iraq, including an area close to Basra (*n* = 9) (560 km South of Baghdad) and in Maysan (*n* = 26) (400 km South of Baghdad). The middle region of Iraq (Baghdad and Babylon) is characterized by a hot and arid climate, while the climate in the southern part of Iraq (Basra and Maysan regions) close to the marshes is hot and more humid (https://www.accuweather.com/en/iq/national/satellite). Samples were shipped to a private company (Deoxi Biotecnologia, http://www.deoxi.com.br/) for genotyping using the Illumina Bovine HD Genotyping BeadChip (700K) (http://www.illumina.com). The geographic location and global positioning system (GPS) coordinates of the Iraqi cattle samples can be found at** Supplementary Table S2A, B**. Aerial distances in kilometers between sampling sites were calculated using the geographic information system (GIS) ArcGIS^®^ software Esri (www.esri.com).

### SNP Genotyping

Ninety-five samples of Iraqi breeds (Rustaqi, *n* = 60; Jenoubi, *n* = 35) were genotyped with the Illumina Bovine HD Genotyping BeadChip (http://www.illumina.com) including 777,962 SNPs mapped to the UMD 3.1; please see section of Data Accessibility for more details (EVA: PRJEB32975; Datadryad.org:dryad.t35r32q). High-density SNP data for references cattle breeds, Holstein-Friesian (European taurine, *n* = 30), Jersey (European taurine, *n* = 32), Nellore (Asian zebu, *n* = 35), Gir (Asian zebu, *n* = 30), Sahiwal (Asian zebu, *n* = 13), EASZ (East African Short horn zebu, *n* = 30), Sheko (East African zebu × taurine cross, *n* = 18), Ankole (East African, *n* = 25), Adamawa Gudali (West African zebu, *n* = 25), Muturu (African taurine, *n* = 12), Red Bororo (West African zebu, *n* = 22), and N’Dama Guinea (African taurine, *n* = 24) were obtained from Bahbahani et al. (2017).

### Data Analysis

Autosomal SNP datasets were prepared using R (https://www.r-project.org) and PLINK 1.9 (Purcell et al., 2007) software. SNPs with minor allele frequency (MAF) of less than 5% (–maf 0.05) and missing genotype data higher than 0.05% (–geno 0.05) were excluded from the analysis. It removed 63,451 variants and 34,387 markers, respectively, leaving 680,124 SNPs for analysis. Also, one sample from Rustaqi, EASZ, and Red Bororo breeds were removed due to their low call rate (<95%).

### Estimation of the Level of Genetic Diversity

To assess the level of genetic diversity, the mean of expected heterozygosity (*He*) and observed heterozygosity (*Ho*) were computed using PLINK 1.9 (Purcell et al., 2007). The genetic diversity was estimated for each population of Iraqi indigenous cattle and several breeds of references. These breeds are Iranian breeds (Sarabi, Kurdi, Taleshi, Pars, Sistani, and Najdi), East Asian native cattle [from Vietnam, Myanmar, Bangladesh, Bhutan, Korea (Hanwoo), Japan (Polled), and Mongolia (MON)], and African breeds [N’Dama Guinea and Sheko (Ethiopian breed)]. Additionally, two important main breeds that represent the main lineage of cattle were used: Holstein-Friesian (*Bos taurus*) and Nellore (*Bos indicus*) (Uzzaman et al., 2014; Karimi et al., 2016; Yonesaka et al., 2016). Furthermore, *He* was assessed under the assumption of Hardy–Weinberg equilibrium, and *Ho* was averaged over loci (Nei, 1978).

### Principal Component Analysis (PCA)

The PLINK 1.9 software was used for principal component analysis (PCA) (Purcell et al., 2007). The autosomal data of the two indigenous Iraqi breeds (Rustaqi and Jenoubi) and several reference breeds [European taurine (Holstein-Friesian, Jersey), Asian zebu (Nellore, Sahiwal, and Gir), African zebu and their crossbreed (East African Shorthorn zebu (EASZ), Sheko, Ankole, Adamawa Gudali, and Red Bororo], and African taurine (Muturu and N’Dama Guinea) were used. The plotting of the PCA results was done using the Genesis software version (0.2.6b) (https://github.com/shaze/genesis).

### Admixture

In order to estimate the ancestry and the genetic structure of the Iraqi cattle population, we used Admixture version 1.3.0 (Alexander et al., 2015) using the following breeds: Rustaqi, Jenoubi, Holstein-Friesian, Jersey, Nellore, Sahiwal, Gir, Brahman, EASZ, Sheko, Ankole, Adamawa Gudali, Red Bororo, and N’Dama Guinea. The analysis was conducted at genome-wide autosomal level, first with Iraqi breeds and four reference breeds (Holstein-Friesian, N’Dama Guinea, Sheko, and Nellore) and then including the entire set of breeds. We performed K = 2 to K = 10 as ancestral modes in order to identify the optimal number of ancestral populations by detecting the lowest value of cross-validation error. We plot our admixture results using the Genesis software (version 0.2.6b) (http://www.bioinf.wits.ac.za/software/genesis/).

#### Positive Candidate Signature of Selection

To construct haplotype files for signature selection analysis, haplotype data of Iraqi breeds (Rustaqi and Jenoubi) and other cattle references were reconstructed by phasing the genotyped SNPs using the SHAPEIT software (v2.8 37) (O’Connell et al., 2014).

### *Rehh* (*iHS* and *Rsb*) Analysis

Identification of signature selection was based on the extended haplotype homozygosity (EHH) tools, using the Rehh package in R. Two analyses were performed (i) based on within-population statistics using Integrated Haplotype Score (iHS) (Voight et al. 2006); and (ii) relative integrated EHH of a site between populations (Rsb) (Sabeti et al., 2002). The *iHS* test was applied to Rustaqi and Jenoubi. *Rsb* test was conducted between i) Jenoubi and Rustaqi and ii) between each Iraqi cattle (Jenoubi and Rustaqi) with three reference breeds (Holstein-Friesian, N’Dama Guinea, and Nellore). The standardized *Rsb* and *iHS* values were normally distributed, so a *Z*-test was applied to identify statistically significant SNPs under selection. One-sided upper-tail *P*-values were derived as 1 − Φ (*Rsb*) from the Gaussian cumulative density function Φ. For Iraqi breeds, we set a threshold of −log_10_ *P*-value = 4 and −4 for the *iHS* test and a threshold of −log_10_ *P*-value = 5 and −5 for the *Rsb* test for the candidate regions. All annotated genes within the region were considered as candidate changes. Then, we examined commonly detected *iHS* and *Rsb* genes for the Iraqi cattle (Jenoubi and Rustaqi) as well as the *Rsb* results of Iraqi cattle and the three reference breeds. A Venn diagram online tool (http://bioinfogp.cnb.csic.es/tools/venny/index.html) was used to check the overlap of candidate genes (Oliveros, 2015).

### Fst Analysis

Inter-population Wright’s *Fst* analyses were conducted between the two Iraqi cattle breeds. *Fst* summarizes the genetic differentiation among populations, through estimation of the allele frequency between populations relative to the total variance of these populations (Wright, 1951; Holsinger and Weir, 2009). The calculation was performed on sliding windows of 60 SNPs, overlapping by 30 SNPs. The above 0.2 of the distribution of *Fst* values was arbitrarily chosen as the significant threshold.

### Gene Function and Gene Pathway Identification Within Candidate-Selected Regions

Gene identification was based on the database of Ensembl *Genes 91*—*Bos taurus* genes (UMD 3.1) using the BioMart tool (http://www.ensembl.org/biomart). PANTHER 11.0 (http://www.pantherdb.org/) and the Enrichr (http://amp.pharm.mssm.edu/Enrichr/) tools were used to explore protein families, molecular functions, biological processes, cellular components, and pathways (Chen et al., 2013; Kuleshov et al., 2016; Mi et al., 2017). Moreover, the list of bovine quantitative trait locus (QTL) regions was downloaded from http://www.animalgenome.org/cgi-bin/QTLdb/index (Hu et al., 2016). Up-to-date information for some specific genes annotation was sourced from Gene Cards (http://www.genecards.org/) and most recent literature, integrating Google Scholar (https://scholar.google.com). To determine over-represented ontology terms for candidate genes following *Fst* analysis, we used DAVID version 6.7 (https://david.ncifcrf.gov/), which detects enriched functional terms (Huang da et al., 2009a; Huang da et al., 2009b) with an enrichment score of 1.3, equivalent to the Fisher exact test *P* = 0.05, as the significant threshold.

## Results

### Genomic Diversity

The highest values of mean *He* and *Ho* were found in Rustaqi and Sheko breeds, while the lowest values were obtained in N’Dama and Nellore (**Table 1** and **Supplementary Figure S2**). In particular, mean *He* and *Ho* were 0.37 and 0.36 for Rustaqi, respectively, while they were 0.32 (*He* and *Ho*) in Jenoubi. Our findings indicate that Iraqi breeds possess significant diversity compared to Asian (e.g., Korean Hanwoo), African (e.g., N’Dama), and European breeds (e.g., Holstein-Friesian) (**Supplementary Tables S3**, **S4**, and **S5**).

**Table 1:** Number of animals, mean of expected heterozygosity (*He*), and observed heterozygosity (*Ho*).

Breed	n	*He* (Mean/SD)	*Ho* (Mean/SD)
Jenoubi	35	0.32/ ± 0.15	0.32/ ± 0.16
Rustaqi	59	0.37/ ± 0.12	0.36/ ± 0.13
Holstein-Friesian	30	0.31/ ± 0.17	0.31/ ± 0.19
Nellore	35	0.22/ ± 0.18	0.23/ ± 0.19
N’Dama Guinea	24	0.23/ ± 0.19	0.23/ ± 0.20
Sheko	18	0.36/ ± 0.12	0.37/ ± 0.15

### Genetic Structure

**Figure 2:**
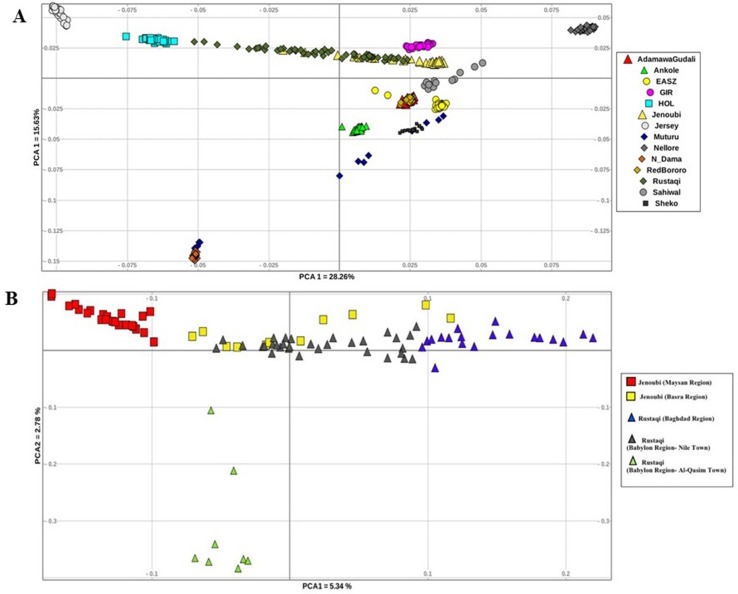
Plots of principal component analysis. **(A)** PCA 1 versus PCA 2 analysis of Iraqi cattle and reference breeds. **(B)** PCA 1 versus PCA 2 analysis of Iraqi cattle (Rustaqi and Jenoubi).

The genetic structure across and within breeds was first assessed using PCA. We conducted two analyses, first between Iraqi breeds and the reference breeds, and then within Iraqi breeds only. For all the breed analysed (**Figure 2A**), PC1 accounts for 28.26% of the total variation. It separates the Asian reference zebu population (Nellore) from the taurine breed (Holstein-Friesian); between the two, we do find the Rustaqi animal to be closer to taurine Holstein-Friesian, and the Jenoubi animals to be closer to the indicine Nellore. PC2, which accounts for 15.63% of the total variation, separates the African zebu breeds (Ankole, Sheko, Adamawa Gudali, and EASZ) and the African taurine (N’Dama and Muturu) from the other cattle populations (non-African breeds). The second PCA implemented for the Iraqi breeds only (Rustaqi and Jenoubi) reveals substructuring within each breed (**Figure 2B**). The first component, which accounts for 5.34% of the total variation, separates the Jenoubi animals sampled in the Maysan region from Rustaqi sampled around Baghdad, while Jenoubi from the Basra region and Rustaqi animals sampled around Babylon region are positioned between these two populations. The second component, which explains 2.78% of the total variation, separates Rustaqi animals from Al-Qasim town (Babylon region) from the other animals.

### Admixture

**Figure 3:**
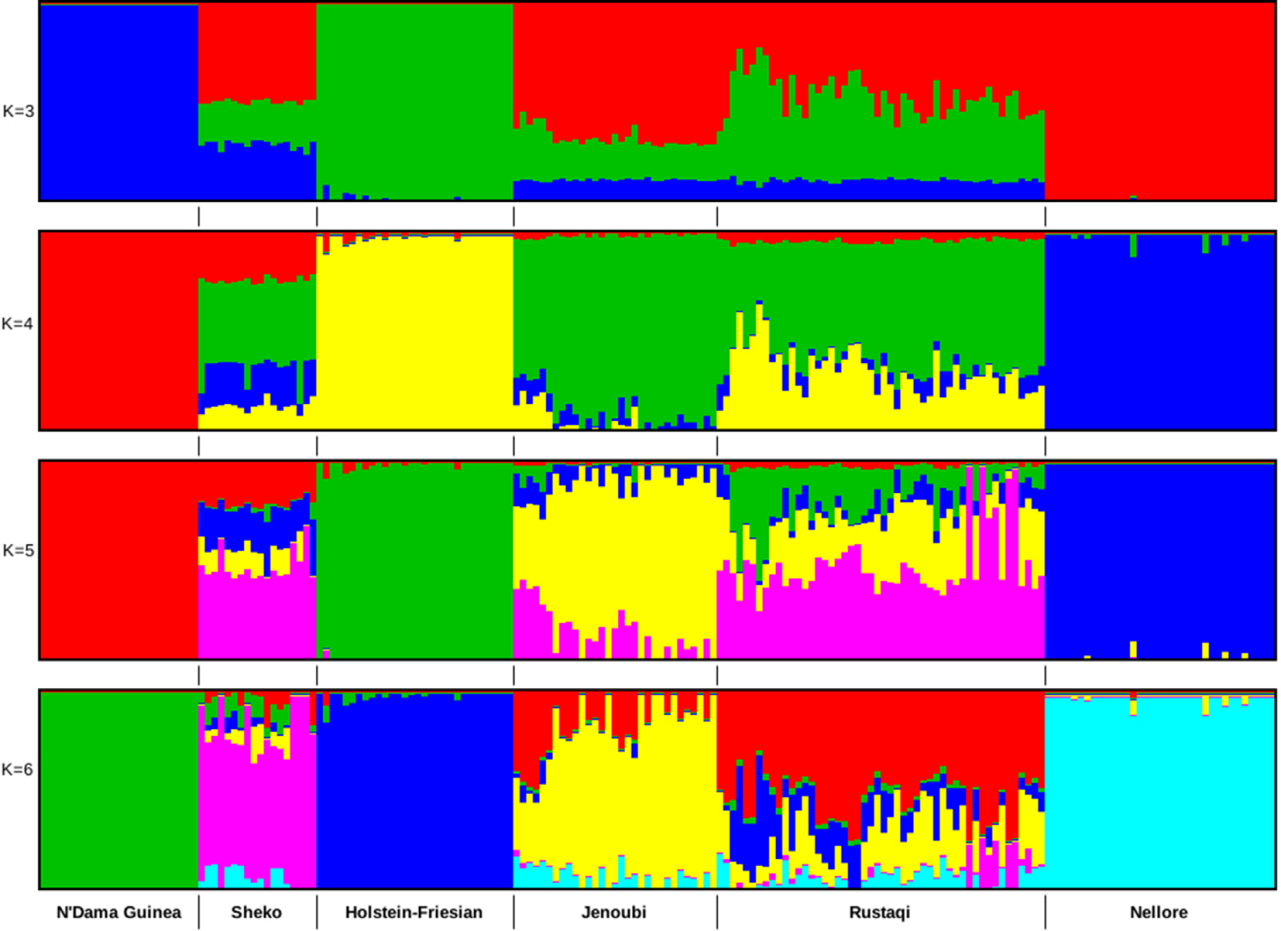
Clustering assignments of five cattle populations based on ADMIXTURE 1.3.0 (Alexander et al., 2015) for inferred *K* values ranging from 2 to 6. Each individual is represented by a single vertical line divided into *K* colored segments, where *K* is the number of ancestral populations.

Admixture analysis was based on 680,124 SNPs after QC. We performed two admixture analyses: first among Iraqi cattle and four main cattle reference breeds (European taurine Holstein-Friesian, Asian zebu Nellore, African crossbreed Sheko, and African taurine N’Dama Guinea) and then including also Jersey, Sahiwal, Gir, East African Shorthorn zebu (EASZ), Ankole, Adamawa Gudali, and Red Bororo. In the first analysis, the selected breeds are representative of the main lineages of cattle (European taurine, African taurine, zebu, and Asian zebu). The optimal number of clusters was here defined as *K* = 4 (it has the lowest cross-validation value). As shown in **Figure 3**, for ancestry *K* = 3, we do observe in Rustaqi and Jenoubi an European taurine and an African taurine shared ancestry as well as an Asian indicine one, for the latter higher in Jenoubi than in Rustaqi. At *K* = 4, shared ancestry with Holstein-Friesian is observed in Rustaqi but much less so in Jenoubi. Shared ancestries with the Nellore and African cattle are present in both Iraqi breeds, but these are low. At *K* = 5 and *K* = 6, both Rustaqi and Jenoubi show admixed background, however, less so for the former than the latter. For the second analysis, the optimal number of clusters was defined as *K* = 7 (**Supplementary Figure S3**). The results obtained here support the previous admixture results with more zebu ancestry in Jenoubi and more taurine ancestry in Rustaqi.

### Genetic Signature of Positive Selection in Iraqi Breeds

**Figure 4:**
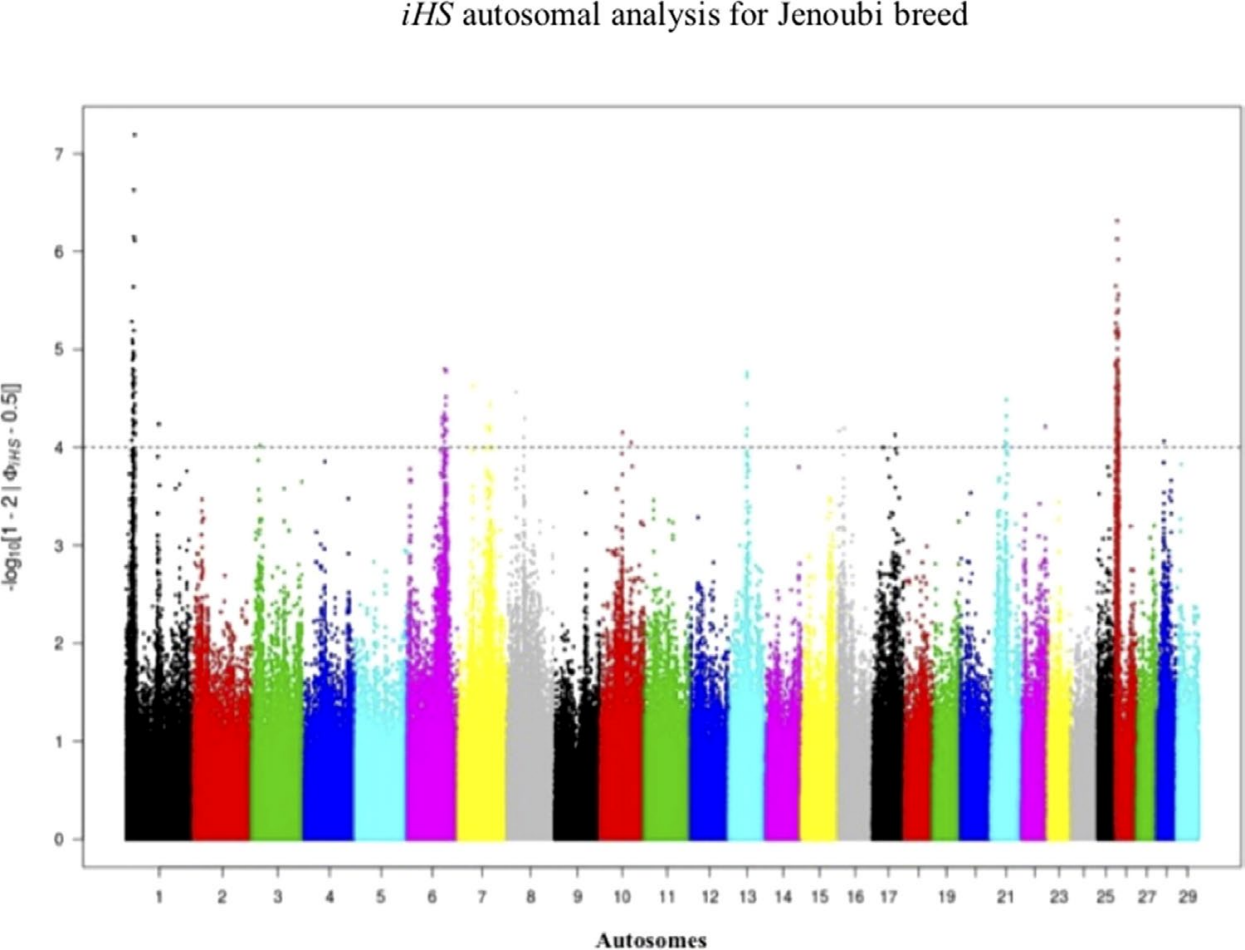
Manhattan plot of genome-wide *iHS* autosomal analysis for Jenoubi. Significance threshold (dashed line) set at −log_10_ > 4.

**Figure 5:**
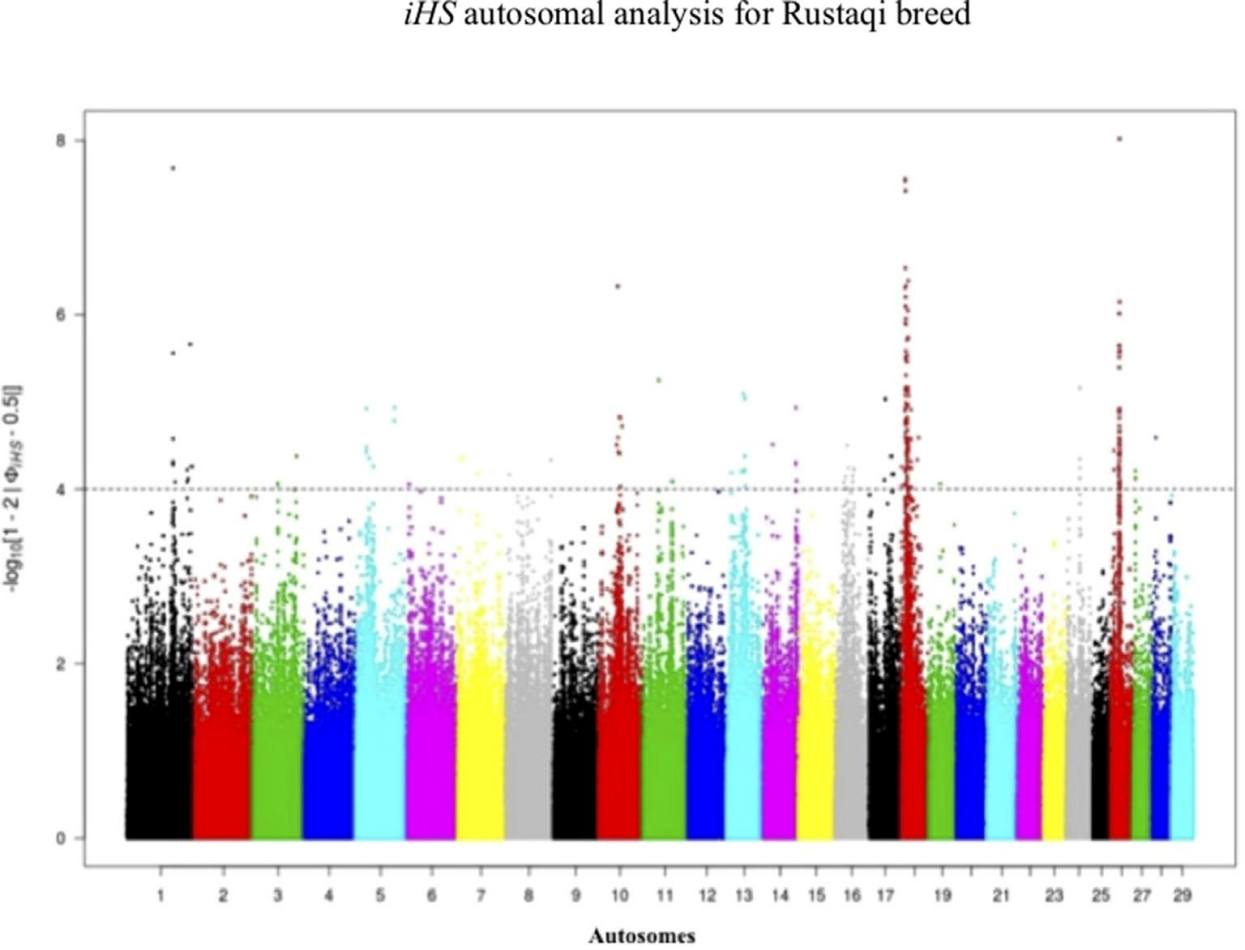
Manhattan plot of genome-wide *iHS* autosomal analysis for Rustaqi. Significance threshold (dashed line) set at −log_10_ > 4.

Footprints of selection for Iraqi breeds (Jenoubi and Rustaqi) were analyzed using *iHS* and *Rsb* statistics, based on extended haplotype homozygosity (EHH). Results are presented in **Figures 4** and **5**. In Jenoubi, we observe the strongest evidence of selection on BTA1 with an *iHS* score of −5.40 and on BTA26 with *iHS* score of −5.0. Several genes are present within these regions, on BTA1 *NCAM2*, *TMPRSS15*, and *CHODL* and on BTA26 *PRKG1*. In Rustaqi, we observe the strongest evidence of selection on BTA1 with *iHS* score of −5.60 and on BTA18 with *iHS* score of −5.03. Genes present within these significant regions include on BTA1 *PPM1L* and *IGSF5* and on BTA18 *PLCG2*, *CDH13*, *NOVEL*, *OSGIN1*, *TLDC1*, *CRISPLD2*, *IRF8*, *JPH3*, *KLHDC4*, *SLC7A5*, *CA5A*, *BANP*, *GALNS*, *CBFA2T3*, *ABCC12*, *ZNF423*, and *LPCAT2*. The Jenoubi breed has 13 candidate-selected regions derived and six ancestral, compared with 11 and 16, respectively, for Rustaqi (**Supplementary Figures S4** and **S5**).

**Figure 6:**
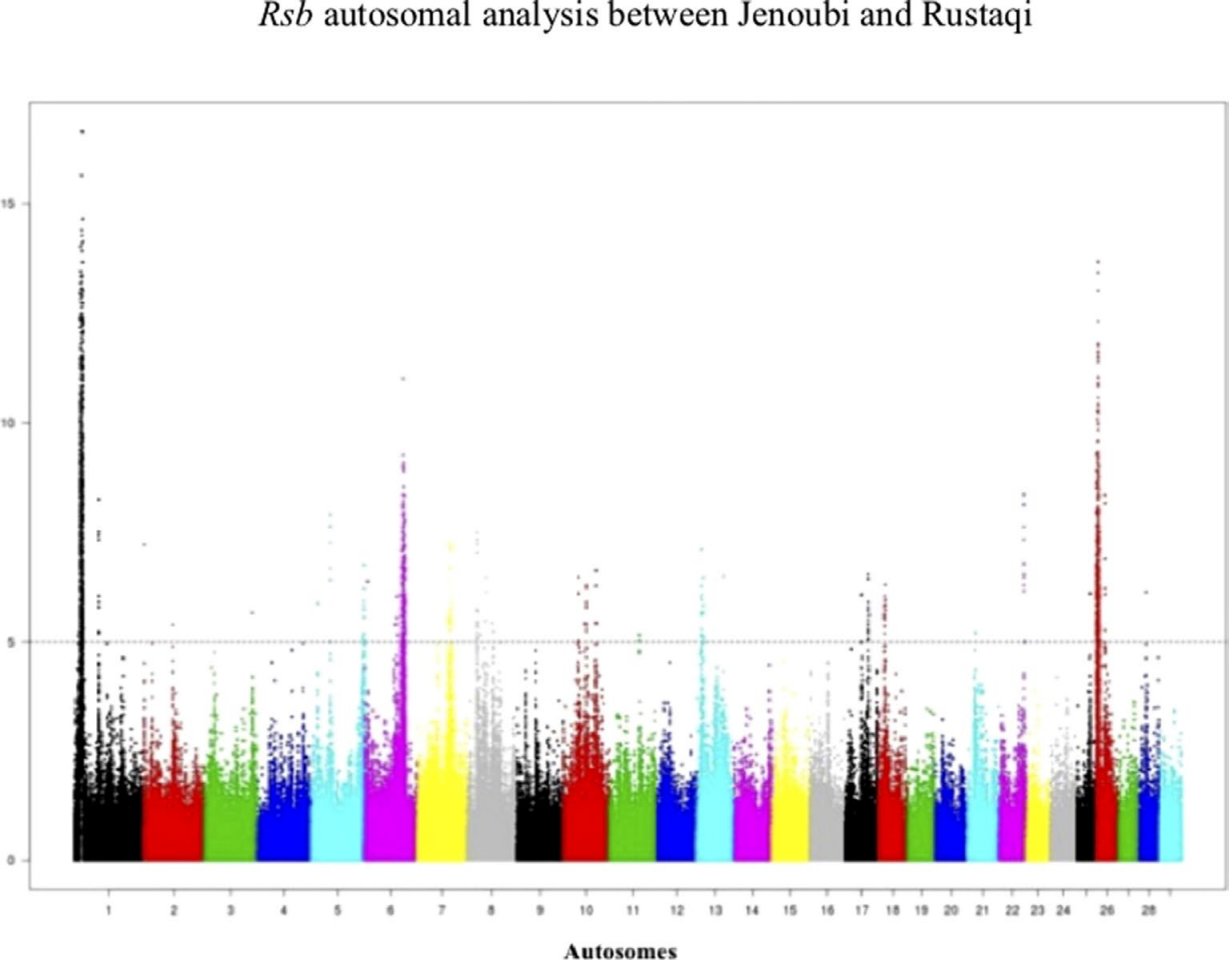
Manhattan plot of the genome-wide *Rsb* autosomal analysis between Jenoubi and Rustaqi. Significance threshold (dashed line) set at −log_10_ > 5.

*Rsb* results between Rustaqi and Jenoubi show a total of 209 SNPs in Jenoubi and 236 in Rustaqi above the significant threshold. The *Rsb* plots show strong signals of positive selection on BTA1, BTA6, BTA7, BTA8, BTA10, BTA17, BTA22, and BTA26 in Jenoubi, and on BTA1, BTA5, BTA13, BTA18, and BTA26 in Rustaqi (**Figure 6**).

### Candidate Genes at Genomic Regions

Candidate regions under positive selection in Jenoubi include 24 annotated genes (14 genes following *iHS* analysis, 17 genes following *Rsb* analysis, and 7 genes present in genome region commonly identified in both tests), while candidate regions under positive selection in Rustaqi include 45 annotated genes (43 genes following *iHS* analysis, 3 genes following *Rsb* analysis, and 1 gene present in genome region commonly identified in both tests); see **Supplementary Table S6**.

**Figure 7:**
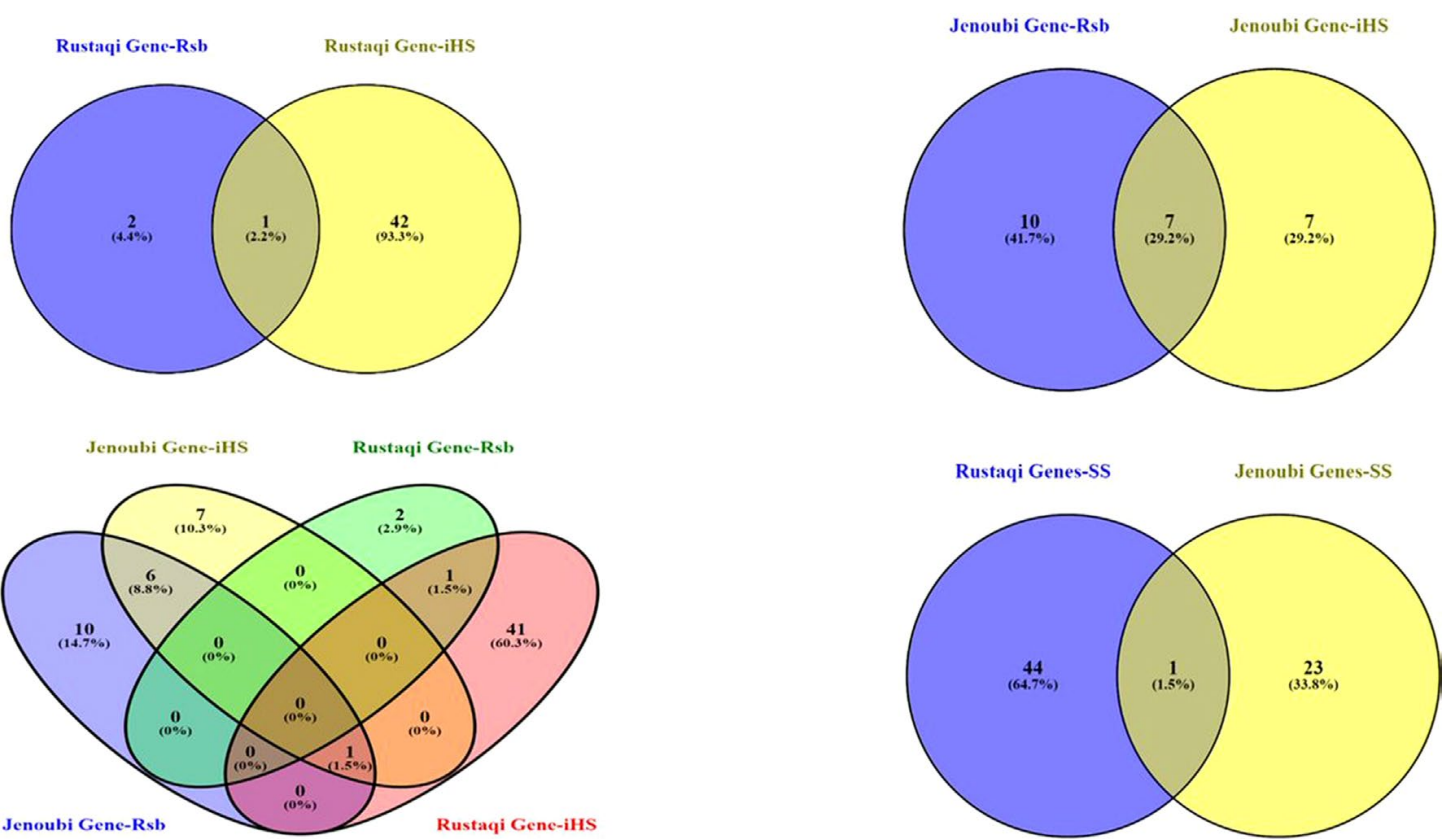
Venn diagram showing the unique and shared genes found within candidate signature selection regions in Jenoubi and Rustaqi. (SS: signature selection genes of iHS&Rsb)

So a total of 68 annotated genes are present within the two Iraqi breeds at the candidate regions defined by the *iHS* and *Rsb* analyses (see **Figure 7** for a Venn diagram showing the number of unique and shared genes found within candidate signature selection regions). The BTA18 regions have the largest number of genes (18 genes), followed by BTA6 with 10 genes, and finally BTA26 with 8 genes.

Overall, the results of genes functions/annotation analysis reveal 19 genes related to the immune system response; 5 genes for Jenoubi, 15 genes for Rustaqi, and 1 overlapping gene between Jenoubi and Rustaqi breeds. This gene is *PRKG1* associated with tick resistance in cattle (Mapholi et al., 2016; Vajana et al., 2018). These immune response genes were involved in both the acquired and innate immune responses, for example, *IRF8* (Rustaqi; BTA18) and *ABCC2* (Rustaqi; BTA26) linked to the acquired immune response to protozoan and bacterial infections (Giagu, 2016) and gastrointestinal nematodes (Li et al., 2011), respectively; *PARM1*, an innate immune response gene (Jenoubi breed; BTA6), associated with anti-apoptotic activity especially during fertility stage (Cochran et al., 2013); and *ATG7* (innate response gene, Jenoubi; BTA22), linked to the autophagy process (Aboelenain et al., 2015).

The remaining genes are related to other environmental adaptation or production characteristics. For instance, *SLC24A3* (Rustaqi breed; BTA13) is related to fertility traits (Moran et al., 2017), and *NCAM2* (Jenoubi, BTA1) is linked to fat, protein, and milk yield (Venturini et al., 2014). **Supplementary Table S7** summarizes the log (*P*-value) of the most significant SNPs within the different significant regions in both breeds. In Jenoubi, the most significant SNPs (*n* = 58 SNPs, maximum SNP log *P*-value = 13.01; BTA26) are within the *PCDH15* gene region involved in the maintenance of the integrity of the intestinal membrane. Then, NCAM2 (*n* = 20 SNPs, maximum SNP log *P*-value = 12.95, BTA1) and *TMPRSS15* (*n* = 58 SNPs, maximum SNP log *P*-value = 12.93, BTA1) are linked to fat, protein, and milk yield.

In Rustaqi, the most significant SNPs (*n* = 25 SNPs, maximum SNP log *P*-value = 8.01; BTA18) are within the *DNMBP* gene region. This gene has been shown to contribute the milk-fat composition (Buitenhuis et al., 2014). Last but not least, the highest number of significant SNP value found in both Rustaqi and Jenoubi is at *PRKG1* (*n* = 25 SNPs, maximum SNP log *P*-value = 7.93; BTA26).

**Figure 8:**
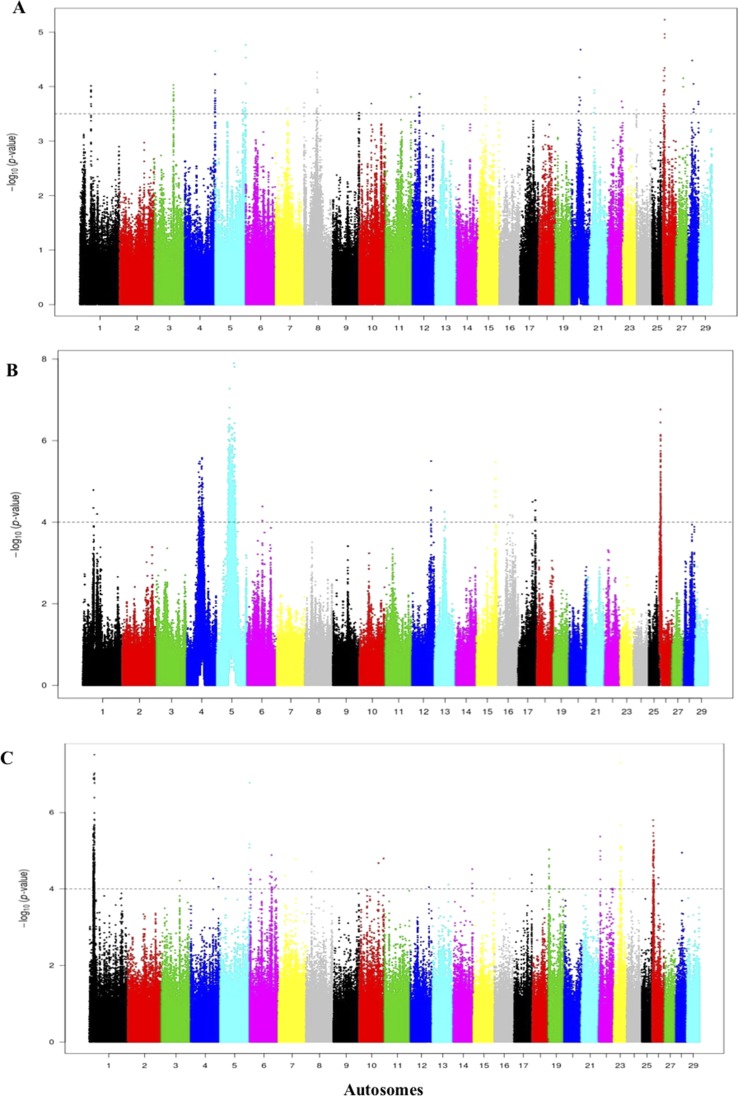
Manhattan plot of the genome-wide *Rsb* autosomal analysis between Jenoubi versus **(A)** Holstein-Friesian, **(B)** Nellore, and **(C)** N’Dama, respectively. Significance threshold (dashed line) set at −log_10_ > 3.5, 4, and 4, respectively.

For the comparisons of the Jenoubi with Holstein-Friesian, Nellore, and N’Dama, we used a threshold for the *Rsb* of >3.5, 4, and 4, respectively (**Figures 8A–C**). We identified 161, 100, and 272 candidate regions for the Jenoubi *Rsb* comparison with Holstein-Friesian, Nellore, and N’Dama, respectively. The most significant SNP values are 5.3 on BTA26 (Jenoubi versus Holstein-Friesian), 7.8 on BTA5 (Jenoubi versus Nellore), and 7.6 on BTA1 (Jenoubi versus N’Dama).

A total of 38 candidate annotated genes are present in significant Jenoubi versus Holstein-Friesian *Rsb* regions. Among these, four candidate genes overlap with previous Iraqi cattle analysis (*iHS* and *Rsb*). These candidate genes are *ATG7* (autophagy control), *PRKG1* (tick resistance), *PCDH15* (maintenance of intestine membrane), and *TMEM132B* (control of brain physiology). The remaining 34 genes were considered to be new genes not identified in our previous analysis of Iraqi breeds. Thirteen genes are included in the Jenoubi–N’Dama Guinea comparison, including eight genes overlapping with the Iraqi breeds analysis (*iHS* and *Rsb*) (e.g., *NCAM2*, *TMPRSS15*, *PCDH15*, *PRKG1*, and *FOCAD*). Only one gene *PCDH15* is present for *Rsb* Jenoubi versus Nellore analysis. (**Supplementary Table S8**).

**Figure 9:**
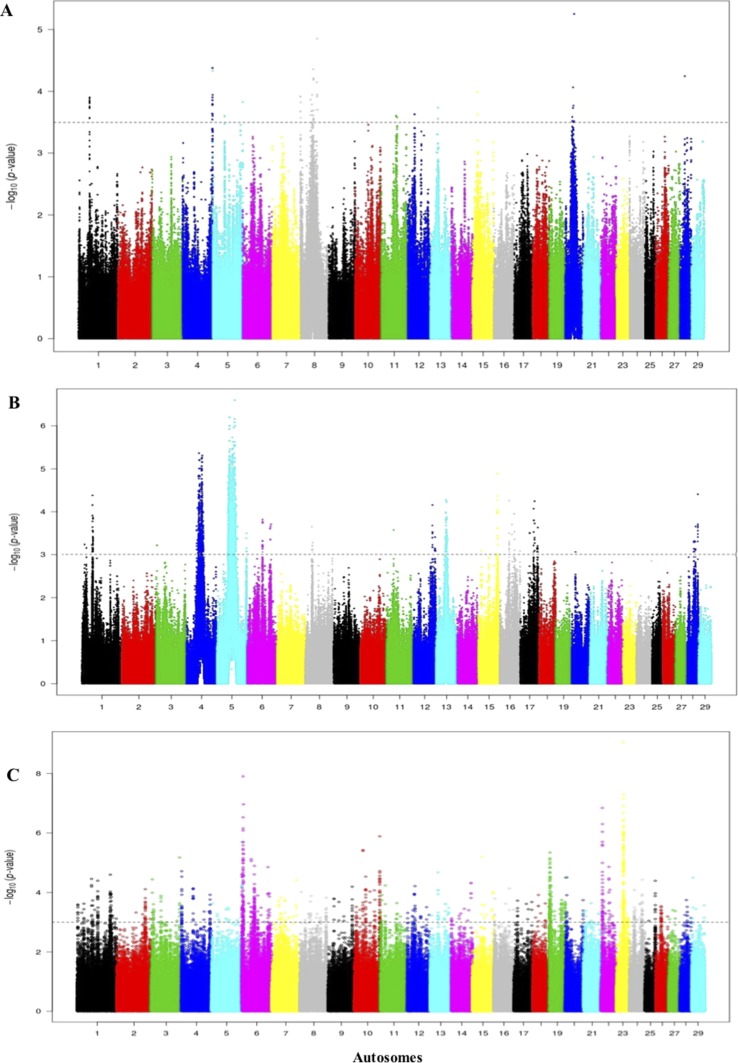
Manhattan plot of the genome-wide *Rsb* autosomal analysis between Rustaqi versus **(A)** Holstein-Friesian, **(B)** Nellore, and **(C)** N’Dama, respectively Significance threshold (dashed line) set at −log_10_ > 3.5, 3, and 3.5, respectively.

The threshold for the Rustaqi *Rsb* analysis was >3.5, 3, and 3.5 for the Holstein-Friesian, Nellore, and N’Dama, respectively. Fifty-seven, 18, and 98 candidate regions were identified from the Rustaqi breed versus Holstein-Friesian, Nellore, and N’Dama analyses, respectively (**Figures 9A–C**). Additionally, the strongest SNP values were 5.3 on BTA20 (Rustaqi versus Holstein-Friesian), 6.8 on BTA5 (Rustaqi versus Nellore), and 7.2 on BTA6 (Rustaqi versus N’Dama). While 12 genes are present in the comparison Rustaqi versus Holstein-Friesian, none overlapped with previous genes identified in the Iraqi analysis (*iHS* and *Rsb*). *Rsb* analysis of Rustaqi against Nellore uncovered five genes, again with no shared genes with our previous Iraqi analysis (*iHS* and *Rsb*). *Rsb* Rustaqi versus N’Dama Guinea found 26 genes, with one gene (*CD96*) previously identified in Iraqi breeds (**Supplementary Table S9**).

### Gene Ontology Analysis

The PANTHER analysis of the biological processes for Jenoubi (iHS genes) reveals the following significant categories: biological regulation, molecular function, cellular components, protein class, and pathways (**Supplementary Figure S6**; **Supplementary Table S10A–D**). On the other hand, the Enrichr tool reveals the following biological processes: positive regulation of apoptotic process, sensory perception of light stimulus, and regulation of GTPase activity. Molecular function analysis shows three enriched levels of gene clusters [apoptotic process (GO: 0043065), transmembrane–ephrin receptor activity (GO:0005005), and calcium channel regular activity (GO:0005246)] (**Supplementary Figure S7A** and **B**; **Supplementary Table S11A** and **B**).

The PANTHER analysis for Rustaqi (iHS genes) identifies the same categories (biological regulation, molecular function, cellular components, protein class, and pathway) (**Supplementary Figure S8**). However, the Enrichr tool analysis reveals two enriched clusters: regulation of positive chemotaxis (GO:0050926) and glomerular epithelial cell development (GO:0072310). On the other hand, the results of molecular function analysis indicate one enriched cluster [O-acetyltransferase activity (GO: 0016413)] (**Supplementary Figure S9A** and **B**).

For the *Rsb* analysis, the PANTHER for Jenoubi indicates eight clusters in biological categories: biological regulation (GO:0065007) (5 genes), biological adhesion (GO:0022610) (3 genes), cellular process (GO:0009987) (12 genes), localization (GO:0051179) (6 genes), metabolic process (GO:000 8152) (5 genes), cellular component organization or biogenesis (GO:0071840) (2 genes), multicellular organismal process (GO:0032501) (1 gene), and response to stimulus (GO:0050896) (1 gene). On the other hand, molecular function shows binding (GO:0005488) (6 genes), catalytic activity (GO:00 03824) (3 genes), and transporter activity (GO:0005215) (3 genes). The Enrichr tool reveals the following biological processes: vitamin D metabolic process and growth factor activity. These ontologies and others (cellular components, protein class, and pathway) are further shown at **Supplementary Figures S10** and **11**.

The PANTHER analysis for Rustaqi (*Rsb* analysis) indicates the following two biological process supported with two genes, cellular process (GO:0009987) and metabolic process (GO:0008152), and the biological process binding (GO:0005488) with one gene. The Enrichr analysis identifies three enriched terms: small GTPase-mediated signal transduction (GO:0007264), cell–matrix adhesion (GO:0007160), and positive regulation of hydrolase activity (GO:0051345). On the other hand, the molecular function analysis recognizes one enriched term, Rho guanyl-nucleotide exchange factor activity (GO:0005089).

### *Fst* Candidate Gene Regions

**Figure 10:**
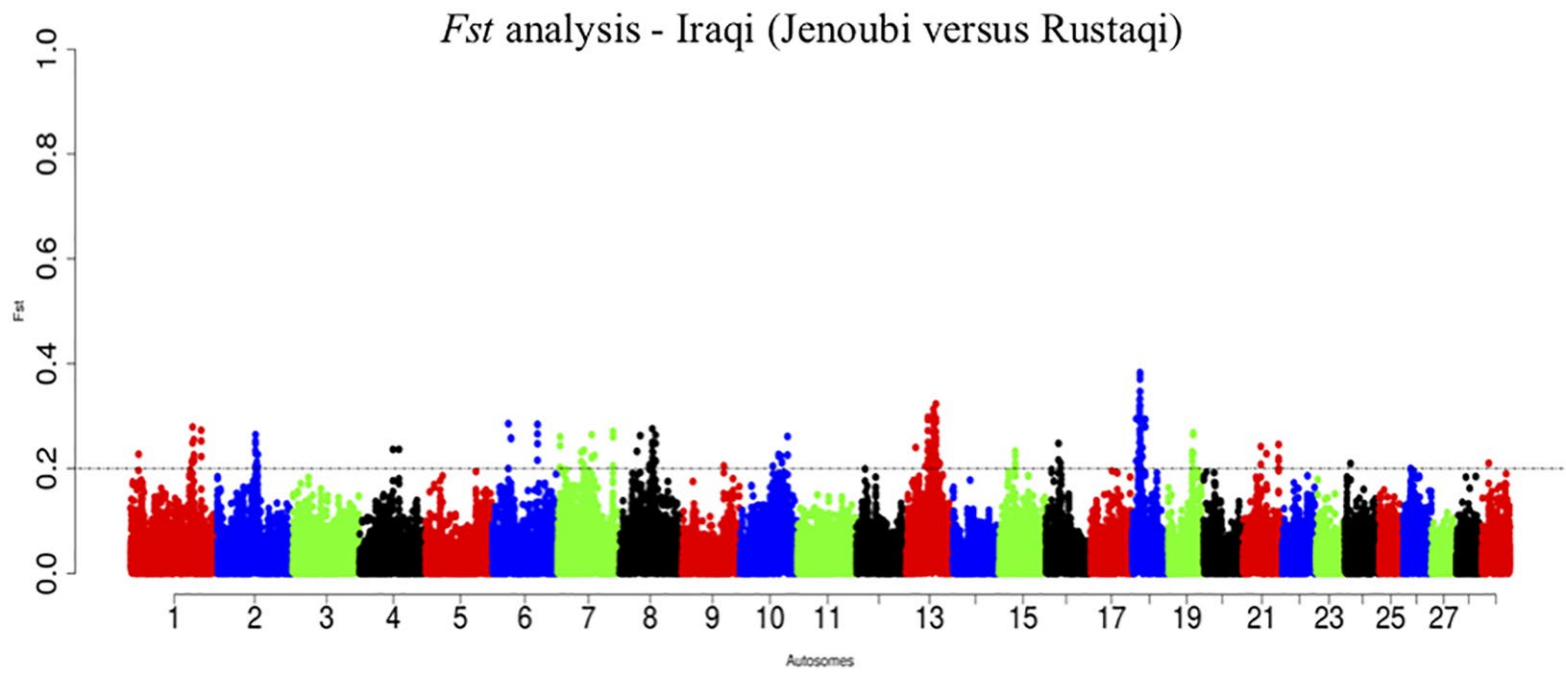
Manhattan plots of the pairwise genome-wide autosomal *Fst* analyses between Rustaqi and Jenoubi for autosomes. The significant thresholds (dashed line) are set at above 0.2 of the *Fst* windows distribution.

The overall genome differentiation of *Fst* values between Iraqi breeds is *Fst* = 0.28 (**Figure 10**). The *Fst* analysis reveals regions with candidate genes differentiated between Jenoubi and Rustaqi (**Supplementary Table S12**). DAVID bioinformatics analysis for *Fst* results shows 16 annotation clusters, but only one of them (metal thiolate and mineral absorption cluster) representing 51 genes has an enrichment score of 4.89, largely above the threshold of 1.3 (*P* = 0.05), with the next cluster, enrichment score 1.29 (metal binding cluster), just below the significant threshold level considered.

## Discussion

In this study, we report at a genome-wide level for the first time the genetic structure, diversity, and candidate signatures of positive selection in two Iraqi cattle breeds, Jenoubi and Rustaqi. At the crossroad of the zebu and taurine centers of domestication, Iraqi cattle may be expected to show high diversity of both taurine and zebu origin. This is confirmed in our study with the presence of both indicine and taurine ancestry in the two breeds, although in different proportions for each of them. Jenoubi cattle are classified as zebu following their humped cattle phenotypes. Our principle component and admixture analyses support such classification, but they also reveal a small proportion of taurine ancestry in their genetic backgrounds. Two factors may have contributed in the shaping of the genetic make-up of Jenoubi. The taurine background within this breed may be corresponding to ancient admixture events from the putative Near East cattle taurine domestication centers, and/or it is the consequence of recent exotic taurine introgression. At K = 3, the Jenoubi taurine background is shared with N’Dama and European taurine. However, at K = 4, the optimal K, the ancestry with European taurine largely disappears. The native habitat of the Jenoubi breed (South-Eastern of Iraq) is away from areas where exotic breeds (e.g., Holstein-Friesian) have been introduced in Iraq in the recent past (Al-Bayatti et al., 2016), supporting ancient taurine introgression events rather than a more recent one from European exotic taurine.

In contrast, with a significant proportion of European ancestry, as revealed in our admixture analysis, recent gene flow from exotic cattle origin likely occurred in Rustaqi. The geographic origin of this breed is central Iraq. It is close to the capital Baghdad, and crossbreeding with exotic taurine might have been driven by the pressures to increase milk production in response to the consumer demands from the city. Admixture analysis indicates also some low zebu background in Rustaqi. Likewise, with the taurine introgression in Jenoubi breed, it may be of ancient origin and the consequence of past trading networks not only between central Iraq and southern Iraq but also further north and south, linking the ancient civilization of the Fertile Crescent and the Indus Valley (Magee et al., 2014).

Interestingly, genetic studies in Anatolian cattle (Anatolian Black, South Anatolian Red, Anatolian Southern Yellow, and Turkish Grey) have also revealed taurine × zebu admixture (Ateş et al., 2014). Also, Karimi et al. (2016) have mentioned that indigenous Iranian cattle from the western part of the country, near the Iraqi border, have more taurine genetic background than southwest Iranian cattle on the south-east border with Iraq, which are more indicine in their genetic background. Our study together with previous ones illustrates the pattern and gradient of zebu and taurine genetic admixture in the region. In terms of genome diversity, the Rustaqi *He* is higher compared with all the Iranian cattle population examined by Karimi et al. (2016). Similarly, comparison of the Jenoubi and Rustaqi with African taurine (N’Dama Guinea), Asian zebu (Nellore), and European Holstein-Friesian shows that Iraqi breeds possess higher genome diversity. It is expected for a crossbred population compared with the non-admixed taurine and zebu population. We also do observe higher genome diversity in Jenoubi and Rustaqi compared with the Sheko, an African zebu × taurine admixed breed (Bahbahani et al., 2017). It may be explained by the closer proximity of Iraqi cattle to the centers of cattle domestication, and therefore the center diversities of domestic cattle compared with the African breeds.

Our signature of selection results in both Iraqi breeds, are suggesting that environmental challenges including diseases pressures have shaped the genomes of Rustaqi and Jenoubi breeds, but not in an identical way, with important differences between the two breeds according to our findings from the *iHS* and *Rsb* analyses.

The *iHS* results in Rustaqi indicate more candidate-selected regions with genes involved in innate and acquired immunities, compared with the results obtained in Jenoubi. Interestingly, among the 14 immune response-related genes unique to Rustaqi, we do find that *OSGIN1* and *CBFA2T3* previously showed to be part to the cattle immune response to mammary gland inflammation (Gilbert et al., 2012; Wang et al., 2012; Osińska et al., 2014). Furthermore, *IRF8* plays an important role against bacterial (e.g., *Salmonella*) and protozoan infections (Gautier et al., 2009; Porto-Neto et al., 2013; Giagu, 2016). The results suggest that the importance of the Rustaqi breed in milk production may have shaped, at least partly, the candidate signatures of selection observed here.

Nevertheless, the *iHS* results in Jenoubi breed have revealed five important immunity genes. *TNFAIP8* and *FOCAD* candidate genes are known to play a role in immune homeostasis and tumor suppression (Hadisaputri et al., 2012; Iwata, 2016). Another crucial gene is *ATG7*, an autophagy gene that contributes to the regulation of the cell death process through elimination of unwanted or dead cells (Aboelenain et al., 2015).

Among the genes identified in both breeds, *PRKG1*, which has been reported previously in two other studies (Mapholi et al., 2016; Vajana et al., 2018), is associated with the tick resistance–tolerance phenotype, a major issue in the pastoral areas of the middle and southern regions of Iraq (Al-Ramahi and Kshash, 2011; Mohammad, 2015). Our study is adding further support to the importance of this gene in relation to disease resistance traits. Similarly, *ABCC2* identified in Rustaqi breed has been related to resistance–tolerance to gastrointestinal nematode parasites infection.

We also identified several regions including genes that may be linked to environmental agro-climatic adaptation in both breeds following *iHS* analysis. Rustaqi animals are raised in a relatively dry and hot environment, and accordingly, adaptation to heat stress may be expected. Here, we do find *UCN3* involved in the genetic control of heat tolerance and oxidative stress, including in Holstein-Friesian cattle (Zheng et al., 2014). Also, in Jenoubi, we identified within candidate-selected regions two genes related to nutrition, *SLC4A4*, which plays a crucial role in the rumen development (Connor et al., 2013), and *EPHA5*, contributing to the improvement of the feed conversion rate from rumination (Santana et al., 2016). This suggests that the breed, largely free grazing, may be particularly adapted to the local availability of feeds.

Although overlap regions are few between Rustaqi and Jenoubi breeds, the same gene pathways may have been under selection pressures in both breeds. For example, *NCAM2*, *TMPRSS15*, and *SLC4A4* within candidate regions in Jenoubi breed have functions related to milk quality and production, with the latter also found under a candidate signature of selection region in Holstein cattle (Li et al., 2010; Venturini et al., 2014). The same regions are not significant in Rustaqi, but here, other genes linked to milk quality and production are found in other significant regions, such as *LPCAT2* and *DNMBP*, two genes linked to protein and fat content in milk (Ogorevc et al., 2009; Buitenhuis et al., 2014; Venturini et al., 2014). Interestingly, we note also the presence in Jenoubi of *PCDH15*, a gene involved in meat quality within a candidate-selected region (Ryu and Lee, 2014).

The outputs of the Enrichr analysis for Jenoubi (*iHS* analysis) indicate that the most enriched cluster among the biological process category is the gene ontology term apoptotic process. Programmed cell death (apoptosis) is part of the immune adaptive response of an organism. In particular, positive regulation of the apoptotic process has been shown to play a role in the immune response of blood cells to trypanosome infection in cattle (Hill et al., 2005), as well as meat quality through elimination of the dead cells, and in maintaining the rumination process of cattle through conserving rumen cells activity (Herrera-Mendez et al., 2006; Connor et al., 2013; Shabtay, 2015). In the Canchim Brazilian beef cattle, the apoptotic process was also among the most enriched clusters (Urbinati et al., 2016). Furthermore, Taye et al. (2017) have mentioned that apoptosis as a response to external stress may be involved in thermotolerance in cattle. Another enriched cluster is sensory perception to light stimulus, which reflects adaptation to vision, one of the cognitive functions of an animal. Such adaptation may be of relevance in particular for outdoor grazing animals (Kim et al., 2017). The GTPase activity cluster found in Jenoubi breed, which plays a significant role in inflammatory reaction following nematode infection (Huang et al., 2007; Kim et al., 2015), and in relation to milk and fertility traits (Kasarapu et al., 2017), has also been found in Yiling yellow Chinese cattle (Ling et al., 2017).

Enrichr results for Rustaqi (*iHS* analysis) indicated biological process related to gene upregulation (clusters regulation of positive chemotaxis and glomerular epithelial cell development) (Pokharel et al., 2018), as well as genes playing a crucial role in defense mechanism against bacterial infection, and growth function processes (Flori et al., 2009; Gautier et al., 2009; Porto-Neto et al., 2013; Giagu, 2016).

PANTHER analysis of Jenoubi *Rsb* results reveals several clusters linked to biological process. For instance, cluster genes of metabolic process are associated with milk production, metabolism of water-soluble vitamins, and regulation of actin cytoskeleton (Raven et al., 2016). Another important cluster is biological regulation including genes involved in rumen and muscle development (Feng et al., 2007; Li et al., 2010). Enrichr analysis identified one enriched category in the biological process cluster (vitamin D metabolic process) and one enriched category in molecular function cluster (growth factor activity). Both clusters may be linked to the health of the animals. For example, vitamin D contributes to the protection of the body from autoimmune diseases with deficiency in vitamin D linked to pathologies, such as osteoporosis and skin or coating diseases (Adorini and Penna, 2008; Bikle, 2014).

Regarding *Rsb* results of Rustaqi breed, the more important ontology term from the Enrichr analysis is the cell–matrix adhesion cluster that regulates tissue construction and cell activity (Lodish et al., 2000). For example, this cluster includes *MYRFL-201*, which protects the myelinated central nervous system, and *DNMBP*, responsible for milk quality traits such as fat composition (Buitenhuis et al., 2014; Koenning, 2015).

Interestingly, among the regions differentiated between the two breeds (*Fst* analysis), DAVID tool identifies the significance cluster metal-thiolate function important for metabolism detoxification activities (e.g., after zinc and copper ingestions) (Richards, 1989). It supports that the two breeds are exposed to different feeds with different toxicity, and they may have responded to such selection pressures accordingly.

In conclusion, we have reported here for the first time at a genome-wide level the genetic structure, diversity, and candidate signatures of positive selection in two Iraqi cattle breeds. Our results support the phenotypic classification of Jenoubi cattle as zebu, and Rustaqi cattle as taurine but with introgression from the other cattle subspecies in each of them. In addition, the results show a significant level of genetic diversity in indigenous Iraqi cattle in line with their history. Genome-wide analysis unravels the genes that play an important role in immunity and other environmental adaptive traits, including in relation to parasitic, bacterial disease challenges, and heat tolerance. This study illustrates the uniqueness of these two indigenous breeds, while the information obtained is expected to help the control of diseases, conservation, management, and utilization of the indigenous Iraqi cattle genetic resources.

## Ethics Statement

The animals used in this study are owned by farmers. Prior to sampling, the objectives of the study were explained to them in their local languages so that they could make an informed decision regarding giving consent to sample their animals. Government veterinary, animal welfare, and health regulations were observed during sampling of the populations analyzed here. The procedures involving animal sample collection also followed the recommendation of directive 2010/63/EU. Collection of blood samples was permitted by the Iraqi Ministry of Agriculture.

## Author Contributions

AA and OH conceived and designed the experiment. AA and OH performed the experiment. AA, AE, and SA-B collected samples. AA analyzed the data. AA and OH wrote the manuscript. All authors have agreed on the contents of the manuscript.

## Funding

We would like to extend our sincere gratitude to the Iraqi Ministry of Higher Education and Scientific Research (MOHESR, Grant 1362), Iraqi cultural attaché, for sponsoring this work

## Conflict of Interest Statement

The authors declare that the research was conducted in the absence of any commercial or financial relationships that could be construed as a potential conflict of interest.
