# Supplementary material for: Genome Analysis Reveals Genetic Admixture and Signature of Selection for Productivity and Environmental Traits in Iraqi Cattle
Source: Front Genet. 2019 Jul 16;10:609. doi: 10.3389/fgene.2019.00609 (PMC6646475; doi:10.3389/fgene.2019.00609)
Supplement: Supplementary file 1 [file Table_1.pdf]

Supplementary Table S1: Breeds and characteristics of Iraqi cattle

**Region**	**Specific Characteristics**	**Weight (kg)**
Rustaqi (Al-Murrani *et al.*, 2003; Al-Bayatti *et al.*, 2016)		
Central Iraq (Baghdad and Babylon provinces)	Brown, black to grey in color, not pigmented skin, small size/male and no hump in female, tolerant to heat stress, endemic diseases and parasites.	450 kg (male)<br>400 kg (female)
Jenoubi (Al-Murrani *et al.*, 2003)		
Southern Iraq<br>(Basra, Maysan)	Red and sometimes pied color	325 kg (male)<br>309 kg (female)
Karradi (Al-Murrani *et al.*, 2003)		
Northern Iraq (Kurdistan)	Mostly black tend to be brown with light spots	300 kg (male)<br>220 kg (female)
Sharabi (Al-Murrani *et al.*, 2003; Maaroof, 2011)		
Northern Iraq (Tigris Valley, North of Mosul)	Black and white color with white line dividing all the body.	400 kg (male)<br>295 kg (female)
