# Supplementary material for: Genome Analysis Reveals Genetic Admixture and Signature of Selection for Productivity and Environmental Traits in Iraqi Cattle
Source: Front Genet. 2019 Jul 16;10:609. doi: 10.3389/fgene.2019.00609 (PMC6646475; doi:10.3389/fgene.2019.00609)
Supplement: Supplementary file 2 [file Table_2.pdf]

Supplementary Table S2a: Gender of each sample within Jenoubi breed, location of population, date of collection, age and GPS information

No. of sample	Gender	Location of population	Age	GPS
1.	Male	Basra\Abo-Alkasib	1,5 year	N: 30.32550<br>E:48.25184
2.	Female	Basra\ Al-Seba	14 months	N: 30.32550<br>E:48.25184
3.	Female	Basra\ Al-Seba	3 years	N: 30.32550<br>E:48.25184
4.	Female	Basra\ Al-Seba	4 years	N: 30.32550<br>E:48.25184
5.	Female	Basra\ Al-Seba	6 years	N: 30.32550<br>E:48.25184
6.	Female	Basra\ Al-Seba	3 years	N: 30.32550<br>E:48.25184
7.	Female	Basra\ Al-Seba	5 years	N: 30.32550<br>E:48.25184
8.	Female	Basra\ Al-Seba	4 years	N: 30.32550<br>E:48.25184
9.	Female	Basra\ Al-Seba	3 years	N: 30.32550<br>E:48.25184
10.	Female	Amara\ Al-Musharah	6 years	N: 31,86904<br>E: 47,45592
11.	Female	Amara\ Al-Musharah	5 years	N: 31,86904<br>E: 47,45592
12.	Female	Amara\ Al-Musharah	11 years	N: 31,86904<br>E: 47,45592
13.	Female	Amara\ Al-Musharah	4 years	N: 31,86904<br>E: 47,45592
14.	Female	Amara\ Al-Musharah	2 years	N: 31,86904<br>E: 47,45592
15.	Male	Amara\ Al-Musharah	1 year	N: 31,86904<br>E: 47,45592
16.	Female	Amara\ Al-Musharah	10 years	N: 31,86904<br>E: 47,45592

17.	Female	Amara\ Al-Musharah	1 year	N: 31,86904<br>E: 47,45592
18.	Female	Amara\ Al-Musharah	7 years	N: 31,86904<br>E: 47,45592
19.	Male	Amara\ Al-Musharah	1 year	N: 31,86904<br>E: 47,45592
20.	Female	Amara\ Al-Musharah	7 years	N: 31,86904<br>E: 47,45592
21.	Female	Amara\ Al-Musharah	2 years	N: 31,86904<br>E: 47,45592
22.	Male	Amara\ Al-Musharah	1 year	N: 31,86904<br>E: 47,45592
23.	Female	Amara\ Al-Musharah	7 days	N: 31,91181<br>E: 47,47033
24.	Female	Amara\ Al-Musharah	15 days	N: 31,91181<br>E: 47,47033
25.	Female	Amara\ Al-Musharah	15 years	N: 31,91181<br>E: 47,47033
26.	Female	Amara\ Al-Musharah	5 years	N: 31,91181<br>E: 47,47033
27.	Female	Amara\ Al-Musharah	4 years	N: 31,91181<br>E: 47,47033
28.	Female	Amara\ Al-Musharah	4 years	N: 31,91181<br>E: 47,47033
29.	Female	Amara\ Al-Musharah	10 years	N: 31,91181<br>E: 47,47033
30.	Female	Amara\ Al-Musharah	8 years	N: 31,91181<br>E: 47,47033
31.	Female	Amara\ Al-Musharah	1,5 year	N: 31,91181<br>E: 47,47033
32.	Male	Amara\ Al-Musharah	1,5 year	N: 31,91181<br>E: 47,47033
33.	Female	Amara\ Al-Musharah	1 year	N: 31,91181<br>E: 47,47033

34.	Female	Amara\ Al-Musharah	1 year	N: 31,91181<br>E: 47,47033
35.	Male	Amara\ Al-Musharah	1,5 year	N: 31,91181<br>E: 47,47033

Supplementary Table S2b: Gender of each sample within Rustaqi breed, location of population, date of collection, age and GPS information.

No. of samples	Gender	Location of population	Age	GPS
1.	Female	Baghdad- Al-Taji	6 years	N\33,3718 E\44,19890
2.	Female	Baghdad- Al-Taji	15 years	N\33,3718 E\44,19890
3.	Female	Baghdad- Al-Taji	5 years	N\33,3718 E\44,19890
4.	Female	Baghdad- Al-Shuaala	7 years	N\ 33.30718 E\ 44.19890
5.	Male	Baghdad- Al-Shuaala	6 months	N\ 33.30718 E\ 44.19890
6.	Female	Baghdad- Abou Graib	5years and 6 months	N\33,36481 E\044,24476
7.	Female	Baghdad- Abou Graib	6 months	N\33,36481 E\044,24476
8.	Female	Baghdad- Abou Graib	4 years	N\33,36481 E\044,24476
9.	Male	Baghdad- Abou Graib	11 months	N\33,36481 E\044,24476
10.	Female	Baghdad- Abou Graib	5 years	N\33,36481 E\44,24476
11.	Female	Baghdad -Al-Fudailia	11 months	N\33.33145 E\44.52449
12.	Female	Baghdad -Al-Fudailia	1 year	N\33.33145 E\44.52449
13.	Female	Baghdad -Al-Fudailia	1 year and 6 months	N\33.33145 E\44.52449
14.	Female	Baghdad -Al-Fudailia	1 year and 2 months	N\33.33145 E\44.52449
15.	Female	Baghdad -Al-Fudailia	2 years	N\33.33145 E\44.52449
16.	Female	Baghdad- Al-Maamil	5 years	N\33.33148 E\44.52449

17.	Female	Baghdad- Al-Maamil	4 years	N\33.33148<br>E\44.52449
18.	Female	Baghdad- Al-Maamil	2 years	N\33.33148<br>E\44.52449
19.	Female	Baghdad- Al-Maamil	6 years	N\33.33148<br>E\44.52449
20.	Female	Baghdad- Al-Maamil	3 months	N\33.33148<br>E\44.52449
21.	Female	Babylon-Al-Sada-Alnasria village	5 years	N\32.32807<br>E\44.52311
22.	Female	Babylon-Al-Sada-Alnasria village	6years and 6 months	N\32.32807<br>E\44.52311
23.	Male	Babylon-Al-Sada-Alnasria village	9 months	N\32.42303<br>E\44.18541
24.	Female	Babylon-Al-Sada-Alnasria village	3 years and 6 months	N\32.42303<br>E\44.18541
25.	Female	Babylon-Al-Sada-Alnasria village	2 years and 6 months	N\32.32807<br>E\44.52311
26.	Female	Babylon-Al-Sada-Alnasria village	5 years and 6 months	N\32.42303<br>E\44.18541
27.	Female	Babylon-Al-Sada-Alnasria village	1year and 6 months	N\32.32807<br>E\44.52311
28.	Female	Babylon-Nile- Taba village	2 years	N\32.32807<br>E\44.52312
29.	Female	Babylon-Nile – Taba village	1 year	N\32.48276<br>E\44.56210
30.	Male	Babylon-Nile – Taba village	2 years	N\32.48276<br>E\44.56210
31.	Female	Babylon-Nile – Taba village	5 years and 6 months	N\32.48276<br>E\44.56210
32.	Male	Babylon- Nile – Taba village	2 years	N\32.48276<br>E\44.56210
33.	Male	Babylon- Nile – Taba village	2 years	N\32.48276<br>E\44.56210
34.	Male	Babylon- Nile – Taba village	2 years	N\32.48276<br>E\44.56210

35.	Male	Babylon- Nile – Taba village	2 years and 6 months	N\32.48276 E\44.56210
36.	Male	Babylon- Nile – Taba village	2 years	N\32.48276 E\44.56210
37.	Male	Babylon- Nile – Taba village	2 years and 6 months	N\32.48276 E\44.56210
38.	Male	Babylon- Nile – Taba village	2 years and 6 months	N\32.48276 E\44.56210
39.	Female	Babylon- Nile – Taba village	3 years and 6 months	N\32.48276 E\44.56210
40.	Female	Babylon- Al-Hamza-Al-Basheia village	4 years	N\32.4370 E\44.4120
41.	Female	Babylon- Al-Hamza-Al-Basheia village	4 years	N\32.4370 E\44.4120
42.	Female	Babylon- Al-Hamza-Al-Basheia village	6 years and 6 months	N\32.4370 E\44.4120
43.	Female	Babylon- Al-Hamza-Al-Basheia village	10 years	N\32.4370 E\44.4120
44.	Male	Babylon- Al-Hamza-Al-Basheia village	2 years	N\32.4370 E\44.4120
45.	Male	Babylon- Al-Hamza-Al-Basheia village	10 months	N\32.4370 E\44.4120
46.	Female	Babylon- Al-Hamza-Al-Basheia village	10 years	N\32.4370 E\44.4120
47.	Female	Babylon- Al-Hamza-Al-Basheia village	5 years and 6 months	N\32.4370 E\44.4120
48.	Female	Babylon- Al-Hamza-Al-Basheia village	5 years	N\32.4370 E\44.4120
49.	Male	Babylon- Al-Hamza-Al-Basheia village	2 years	N\32.4370 E\44.4120
50.	Female	Babylon-Alqasim-Alnakabia village	5 years	N\32.1252 E\44.3928
51.	Female	Babylon-Al-Qasim-Al-Nakabia village	4 years	N\32.1307 E\44.3947
52.	Female	Babylon-Al-Qasim-Al-Nakabia village	8 years	N\32.1252 E\44.3928

53.	Female	Babylon-Al-Qasim-Al-Nakabia village	5 years and 6 months	N\32.1307 E\44.3947
54.	Female	Babylon-Al-Qasim-Al-Nakabia village	3 years	N\32.1307 E\44.3947
55.	Female	Babylon-Al-Qasim-Al-Nakabia village	5 years	N\32.1252 E\44.3928
56.	Female	Babylon-Al-Qasim-Al-Nakabia village	5 years	N\32.1252 E\44.3928
57.	Male	Babylon-Al-Qasim-Al-Nakabia village	2 years and 6 months	N\32.1307 E\44.3947
58.	Male	Babylon-Al-Qasim-Al-Nakabia village	3 years	N\32.1252 E\44.3928
59.	Male	Babylon-Al-Qasim-Al-Nakabia village	2 years and 6 months	N\32.1307 E\44.3947
60.	Male	Babylon-Al-Qasim-Al-Nakabia village	2 years	N\32.1252 E\44.3928
