# Supplementary material for: Genome Analysis Reveals Genetic Admixture and Signature of Selection for Productivity and Environmental Traits in Iraqi Cattle
Source: Front Genet. 2019 Jul 16;10:609. doi: 10.3389/fgene.2019.00609 (PMC6646475; doi:10.3389/fgene.2019.00609)
Supplement: Supplementary file 3 [file Table_3.pdf]

Supplementary Table S3: Comparison of diversity levels among Iraqi_ Iranian cattle.

[Number of animals, mean of expected heterozygosity (*He*), observed heterozygosity (*Ho*) and minor allele frequency (*MAF*)]

**Breed**	**n**	**Genotype**	***He* (Mean)**	***Ho* (Mean)**	***MAF* (Mean)**
**Jenoubi** (Iraqi zebu)	35	HD	0.32	0.32	0.24
**Rustaqi** (Iraqi taurine)	59	HD	0.37	0.36	0.28
Sarabi (Iranian Breed)	19	HD	0.34	-	0.25
Kurdi (Iranian Breed)	7	HD	0.36	-	0.26
Taleshi (Iranian Breed)	7	HD	0.32	-	0.23
Pars (Iranian Breed)	7	HD	0.26	-	0.21
Sistani (Iranian Breed)	9	HD	0.23	-	0.23
Najdi (Iranian Breed)	7	HD	0.31	-	0.31
Kermani (Iranian Breed)	9	HD	0.27	-	0.20
Mazandarani (Iranian Breed)	10	HD	0.32		0.23
Holstein-Friesian (Bos taurus)	30	HD	0.31	0.31	0.23
Nellore (Bos indicus)	35	HD	0.22	0.23	0.16
N'Dama Guinea (Bos taurus)	24	HD	0.23	0.23	0.17
Sheko (Ethiopian_ Bos taurus)	18	HD	0.36	0.37	0.27
Sanga cattle_South Africa (Africana)	42	LD	0.24	-	-
Sanga cattle_South Africa (Nguni)	54	LD	0.28	-	-
Sanga cattle_South Africa (Drakensberger)	47	LD	0.30	-	-
