# Supplementary material for: Genome Analysis Reveals Genetic Admixture and Signature of Selection for Productivity and Environmental Traits in Iraqi Cattle
Source: Front Genet. 2019 Jul 16;10:609. doi: 10.3389/fgene.2019.00609 (PMC6646475; doi:10.3389/fgene.2019.00609)
Supplement: Supplementary file 4 [file Table_4.pdf]

Supplementary Table S4: Comparison of diversity levels among Jenoubi_ East Asian cattle.

[Number of animals, mean of expected heterozygosity (*He*), observed heterozygosity (*Ho*) and minor allele frequency (*MAF*)]

**Breed (*Bos indicus*)**	**n**	***He* (Mean)**	***Ho* (Mean)**	***MAF* (Mean)**
Jenoubi (Iraqi zebu)	35	0.32	0.32	0.24
Vietnamese native cattle (Asian zebu)	30	0.23	0.26	0.20
Myanmar native cattle (Asian zebu)	28	0.26	0.24	0.19
Bangladeshi native cattle (Asian zebu)	30	0.25	0.24	0.18
Bhutanese native cattle (Asian zebu)	28	0.29	0.28	0.21
