# Supplementary material for: Genome Analysis Reveals Genetic Admixture and Signature of Selection for Productivity and Environmental Traits in Iraqi Cattle
Source: Front Genet. 2019 Jul 16;10:609. doi: 10.3389/fgene.2019.00609 (PMC6646475; doi:10.3389/fgene.2019.00609)
Supplement: Supplementary file 5 [file Table_5.pdf]

**Supplementary Table S5: Comparison of diversity levels among Rustaqi_ East Asian.**

[Number of animals, mean of expected heterozygosity (*He*), observed heterozygosity (*Ho*) and minor allele frequency (*MAF*)]

| **Breed (*Bos taurus*)** | **n** | ***He* (Mean)** | ***Ho* (Mean)** | ***MAF* (Mean)** |
|---|---|---|---|---|
| Rustaqi (Iraqi native cattle) | 59 | 0.37 | 0.36 | 0.28 |
| Hanwoo (Korean Native cattle) | 30 | 0.36 | 0.36 | 0.27 |
| Polled (Japanese Native cattle) | 29 | 0.33 | 0.31 | 0.23 |
| MON (Mongol native cattle) | 30 | 0.38 | 0.39 | 0.29 |
