# Supplementary material for: Genome Analysis Reveals Genetic Admixture and Signature of Selection for Productivity and Environmental Traits in Iraqi Cattle
Source: Front Genet. 2019 Jul 16;10:609. doi: 10.3389/fgene.2019.00609 (PMC6646475; doi:10.3389/fgene.2019.00609)
Supplement: Supplementary file 6 [file Table_6.pdf]

Supplementary Table S6: Candidate gene regions with their functions detected by *iHS* and *Rsb* in Iraqi breeds (Rustaqi and Jenoubi)

Chromosome	Candidate gene regions			***iHS* = 4 /- 4 (threshold)**		***Rsb* = 5 /- 5 (threshold)**		**Gene ID**	**Trait association / QTL information (Biological role)**	**Studies**
	Start	*End*	Mb	*Rustaqi*	*Jenoubi*	*Rustaqi*	*Jenoubi*			
1	56722581	56818961	96380	N	N	Y	N	*CD96-201*	Immunological related<br>MHC-class1 gene	(Al Naib *et al.*, 2011)<br>(Wu *et al.*, 2014)
1	14791090	15026555	235465	N	Y	N	Y	*NCAM2*	Healthy related<br>Fat, protein and milk yield. Marbling score of meat.<br>Neurogenesis role (olfactory receptor gene)	(Venturini *et .al*, 2014)<br>(Seong *et al.*, 2016)<br>(Shin *et al.*, 2014)
1	18058709	18207251	148542	N	Y	N	Y	*TMPRSS15*	Healthy related<br>Fat, protein and milk yield.	(Venturini *et al.*, 2014)
1	18208866	18233154	24288	N	Y	N	N	*CHODL*	Healthy related<br>Endocytosis of glycoproteins and exogenous sugar-bearing pathogens	Gene card
1	107241227	107576086	334859	Y	N	N	N	*PPM1L*	Immunological and health related tumour-suppressive and regulate endoplasmic reticulum ER stress	(Flisikowski *et al.*, 2015)<br>(Ren *et al*., 2014)
1	141349995	141401851	51856	Y	N	N	N	*IGSF5*	Immunological related<br>Regulation of leukocytes and immunoglobulins	(Jeong *et al.*, 2015)
3	103353700	103422206	68506	Y	N	N	N	*CFAP57*	Healthy related<br>Formation of protein-protein complexes	Gene card
5	43746464	43872342	125878	N	N	Y	N	*MYRFL-201*	Healthy related<br>Maintain a mature myelinated central nervous system in mice	(Koenning , 2015)
5	33189704	33339728	150024	Y	N	N	N	*PCED1B*	Healthy related<br>Modification of biopolymers on the cell surface	Gene card

Chromosome	Candidate gene regions			*iHS* = 4 /- 4<br>(threshold)		*Rsb* = 5 /- 5<br>(threshold)		Gene ID	Trait association / QTL information<br>(Biological role)	Studies
	Start	*End*	Mb	*Rustaqi*	*Jenoubi*	*Rustaqi*	*Jenoubi*			
5	42837597	43112731	275134	Y	N	N	N	*PTPRR*	Healthy related<br>Regulate a variety of cellular processes. Neuron activity promotion	(Allan *et al.*, 2009)<br>(Hendriks *et al.*, 2009)
5	91835146	92276939	441793	Y	N	N	N	*PIK3C2G*	Healthy related<br>Growth factor.Associated with nervous transmssion	(Flori *et al.*, 2009)<br>(Shin *et al.*, 2014)
6	82560093	82962887	402794	N	Y	N	N	*EPHA5*	Healthy related<br>Behaviour regulated in mice<br>Regulation of insulin secretion.Feed conversion ratio (FCR) in cattle	(Mamiya *et al.*, 2008)<br>(Santana *et al.*, 2016)
6	88182303	88541046	358743	N	Y	N	Y	*SLC4A4*	Healthy related<br>Rumen development and milk Production	(Li *et al.*, 2010)<br>(Connor *et al.*, 2013)
6	88695940	88739180	43240	N	Y	N	Y	*GC*	Healthy related<br>Udder structure, height and cleft	QTL
6	90842934	90985937	143003	N	Y	N	N	*MTHFD2L*	Healthy related<br>Calving interval in dairy cattle. Metabolism of water-soluble vitamins	(Raven *et al.*, 2016)<br>Gene card
6	86169557	86190786	21229	N	N	N	Y	*Novel*	-	-

Chromosome	Candidate gene regions			***iHS* = 4 /- 4**<br>**(threshold)**		***Rsb* = 5 /- 5**<br>**(threshold)**		**Gene ID**	**Trait association / QTL information**<br>**(Biological role)**	**Studies**
	Start	*End*	Mb	*Rustaqi*	*Jenoubi*	*Rustaqi*	*Jenoubi*			
6	87035926	87094952	59026	N	N	N	Y	*SULT1E1*	Healthy related<br>Regulates active oestrogen.<br>Oestrogen metabolic process and regulation of fat cell differentiation.Lipid metabolism	(Ushizawa *et al.*, 2007)<br>(McCabe *et al.*, 2012)<br>(Khatri *et al.*, 2011)<br>(Seong *et al.*, 2016)
6	87179502	87188025	8523	N	N	N	Y	*CSN2*	Healthy related<br>Milk protein production and quality	(Ogorevc *et al.*, 2009)<br>(Rothammer *et al.*, 2013)<br>(Taye *et al.*, 2017)
6	87694412	87705727	11315	N	N	N	Y	*AMBN*	Healthy related<br>Role in differentiation of inner enamel epithelium.Biomineral tissue developemnt	(Perdigao *et al.*, 2004)<br>(Do *et al.*, 2017)
6	89162542	89460195	297653	N	N	N	Y	*ADAMTS3*	Healthy related<br>Fertility trait. Implicated in the processes of luteinization and ovulation process. Udder conformation traits	(Mészáros *et al.*, 2014)<br>(Pausch *et al.*, 2014)<br>(Barthelemy *et al.*, 2018)

Chromosome	Candidate gene regions			***iHS* = 4 /- 4**<br>**(threshold)**		***Rsb* = 5 /- 5**<br>**(threshold)**		**Gene ID**	**Trait association / QTL information**<br>**(Biological role)**	**Studies**
	Start	*End*	Mb	*Rustaqi*	*Jenoubi*	*Rustaqi*	*Jenoubi*			
6	91597122	91723390	126268	N	N	N	Y	*PARM1*	Immunological and healthy related<br>Anti-apoptotic. Daughter pregnancy rate (DPR), heifer conception rate, cow conception rate, productive life, net merit, milk, protein and fat yield	(Cochran, 2013) (Hallé, 2015) (Ortega *et al.*, 2016)
7	71003540	71115619	112079	N	N	N	Y	*CYFIP2*	Healthy related<br>Regulation of actin cytoskeleton	Gene card
7	73928475	73960160	31685	N	N	N	Y	*CCNJL*	-	-
7	35812577	35858617	46040	N	Y	N	N	*TNFAIP8*	Immunological and health related<br>Tumour-suppressive and oocytes growth.	(Hadisaputri *et al.,* 2012) (Zhang,2012) (Iwata *et al.*, 2016)
7	69585066	70027839	442773	N	Y	N	N	*SGCD*	Healthy related<br>Cardiac and skeletal muscles mass.Improve eneregy level in the body	(Bradley *et al.*, 2008) (Martchenko *et al.,* 2018)
7	12206055	12266930	60875	Y	N	N	N	*ADGRE3*	Immunological and health related<br>Play a role in myeloid-myeloid interactions during immune and inflammatory responses.	Gene card
8	23386997	23662561	275564	N	Y	N	Y	*FOCAD*	Immunological related<br>Tumour-suppressive	Gene card
10	44711724	44842190	130466	Y	N	N	N	*GNG2*	Healthy related<br>Involved in signaling echanisms across membranes.	Gene card

Chromosome	Candidate gene regions			***iHS*** **= 4 /- 4 (threshold)**		***Rsb*** **= 5 /- 5 (threshold)**		**Gene ID**	**Trait association / QTL information (Biological role)**	**Studies**
	Start	*End*	Mb	*Rustaqi*	*Jenoubi*	*Rustaqi*	*Jenoubi*			
10	48949618	49750993	801375	Y	N	N	N	*RARA (RORA)*	Immunological and health related<br>Cell-mediated immune response in case of mastitis (CD4 (+)/ T-helper cells mechanisms). Intramuscular fat deposition and composition. Plays an important role in the regulation of lipid homeostasis and the promotion of myogenesis in skeletal muscle. Steroid hormone receptor activity.Fertility trait.	(Ogorevc *et al.*, 2009)<br>(Sevane *et al.*, 2014)<br>(Qanbari *et al.*, 2010)<br>(Bahbahani *et al.*, 2017)<br>(Pandey *et al.*, 2017)
10	51020033	51240077	220044	Y	N	N	N	*MYO1E*	Healthy related<br>Actin-based molecular motors, intracellular movement and membrane trafficking.	Gene card
11	38706659	38928773	222114	Y	N	N	N	*CCDC85A*	-	-
13	36610252	36833259	223007	Y	N	N	N	*MPP7*	-	-
13	39202450	39632869	430419	Y	N	N	N	*SLC24A3*	Healthy related<br>Fertility trait	(Moran *et al.*, 2017)
13	43605579	43610260	4681	Y	N	N	N	*UCN3*	Healthy related<br>Response to heat stress	(Zheng *et al.*, 2014)
16	30656001	30703396	47395	Y	N	N	N	*COQ8A*	Healthy related<br>Lack of this gene led to ataxia in mammals	(Stefely *et al.*, 2016)
16	32381118	32471604	90486	Y	N	N	N	*KIF26B*	Immunological and Healthy related<br>Platelets activation and embryonic kidney development	Gene card

Chromosome	Candidate gene regions			*iHS* = 4 /- 4 (threshold)		*Rsb* = 5 /- 5 (threshold)		Gene ID	Trait association / QTL information (Biological role)	Studies
	Start	*End*	Mb	*Rustaqi*	*Jenoubi*	*Rustaqi*	*Jenoubi*			
16	45621457	45879645	258188	Y	N	N	N	*RERE*	Healthy related<br>Embryonic growth and reproductive development	(Randhawa *et al.*, 2016)<br>(Edea *et al.*, 2018)
17	52565556	52785519	219963	N	N	N	Y	*TMEM132B*	Healthy related<br>control for potential compensatory mechanisms in the brain of mice	(Walser *et al.*, 2012)
18	8427455	8516798	89343	Y	N	N	N	*PLCG2*	Healthy related<br>Prolactin signalling	(Ponsuksili *et al.*, 2012)
18	9512739	10162782	650043	Y	N	N	N	*CDH13*	Healthy related<br>Chest width, Dry matter intake, Residual feed intake	QTL
18	10250180	10263073	12893	Y	N	N	N	*NOVEL*	-	-
18	10294996	10305608	10612	Y	N	N	N	*OSGIN1*	Immunological and Healthy related<br>Oxidative stress response protein that regulates cell death in mammary tissue. Key regulator of both inflammatory and anti-inflammatory molecules.	(Gilbert *et al.*, 2012)<br>(Osińska *et al.*, 2014)<br>Gene card
18	10694237	10706275	12038	Y	N	N	N	*TLDC1*<br>*- Kiaa1609*	Healthy related<br>Neuroprotective against oxidative stress	(Finelli *et al.*, 2016)
18	10985132	11050904	65772	Y	N	N	N	*CRISPLD2*	Immunological related<br>Regulate Endotoxin Function<br>(anti-inflammatory function)	(Cheng *et al.*, 2015)<br>(Wang *et al.*, 2009)

Chromosome	Candidate gene regions			***iHS* = 4 /- 4 (threshold)**		***Rsb* = 5 /- 5 (threshold)**		**Gene ID**	**Trait association / QTL information (Biological role)**	**Studies**
	Start	*End*	Mb	*Rustaqi*	*Jenoubi*	*Rustaqi*	*Jenoubi*			
18	11883376	11905043	21667	Y	N	N	N	*IRF8*	Immunological related<br>Defence response to bacterium, defence response to protozoan, main activity in proliferation and differentiation of immune cells, lacking this genes led to susceptible to Salmonella and Mycobacterial infection, plays an important role for pre-B-cell development	Gautier *et al.,* 2009)<br>(Porto-Neto *et al.,* 2013)<br>((Giagu, 2016)
18	13186023	13260798	74775	Y	N	N	N	*JPH3*	Healthy related<br>Cell surface and intracellular ion mechanism. Skeletal and Muscular System Development and Function in cattle. Meat quality trait (intramuscular fatty acid deposition, and muscle fiber in pigs)	Gene card<br>(Barbisin *et al.*, 2014)<br>(Jeong *et al.*, 2015)
18	13268589	13299788	31199	Y	N	N	N	*KLHDC4*	Healthy related<br>Residual feed intake (RFI). Regulate Conjugated linoleic acid (CLA) in early lactating in cattle liver	(Garbe, 2015)<br>(Ringseis *et al.*, 2016)
18	13341371	13370494	29123	Y	N	N	N	*SLC7A5*	Immunological and Healthy related<br>Glucose Metabolism and Blood-Brain Barrier and Immune Cell Transmigration. Also, embryonic developmentand reproductive functions.Dairy traits	Gene card<br>(Gautier *et al.*, 2011)<br>(Joost et al., 2015)
18	13389828	13412237	22409	Y	N	N	N	*CAER5A*	-	-
18	13425303	13493366	68063	Y	N	N	N	*BANP (BTG3)*	Immunological related<br>T-cell development. Tumour suppressor. Important for fetal muscle growth.	Gene card<br>(Feng *et al.*, 2007)

Chromosome	Candidate gene regions			***iHS* = 4 /- 4** **(threshold)**		***Rsb* = 5 /- 5** **(threshold)**		**Gene ID**	**Trait association / QTL information** **(Biological role)**	**Studies**
	Start	*End*	Mb	*Rustaqi*	*Jenoubi*	*Rustaqi*	*Jenoubi*			
18	14061951	14077096	15145	Y	N	N	N	*GALNS*	Healthy related<br>Effects on Tissues rich in keratan sulfate including cartilage, cornea and heart valve.	Gene card
18	14096667	14174040	77373	Y	N	N	N	*CBFA2T3*	Immunological related<br>Associated with cellular proliferation, lipid metabolism, and innate immunity in dairy cow mammary gland tissue at different stages of lactation.	(Wang *et al.*, 2012)
18	16551061	16610345	59284	Y	N	N	N	*ABCC12*	Healthy related<br>Muscle Mass trait	(Kärst *et al.*, 2011)
18	18041277	18392207	350930	Y	N	N	N	*ZNF423*	Healthy related<br>Residual feed intake, dry matter intake (DMI) and skeletal muscles trait (fat)	(Abo-Ismail *et al.,* 2014)<br>(Guo *et al.*, 2015)
18	23861829	23927174	65345	Y	N	N	N	*LPCAT2*	Healthy related<br>Milk, fat, and protein yield.	(Venturini *et al.*, 2014)
18	39221305	39300130	78825	Y	N	N	N	*PKD1L3*	Healthy related<br>Kidney function	Gene card
21	35415163	35655955	240792	N	Y	N	N	*STXBP6*	Healthy related<br>Meat quality (marbling score).	(Ryu *et al.*, 2014)
22	55944219	56217921	273702	N	Y	N	N	*ATG7*	Immunological and Healthy related<br>Autophagy and hematopoietic stem cell maintenance	(Aboelenain *et al.*, 2015)<br>Gene card
22	56530332	56533155	2823	N	N	N	Y	*TRH*	Healthy related<br>releases mature thyrotropin-releasing hormone	Gene card
24	33386429	33419545	33116	Y	N	N	N	*ANKRD29*	Healthy related<br>Down regulated for adipocytes (intramuscular fat)	(Yu *et al.*, 2009)

Chromosome	Candidate gene regions			*iHS* = 4 /- 4 (threshold)		*Rsb* = 5 /- 5 (threshold)		Gene ID	Trait association / QTL information (Biological role)	Studies
	Start	*End*	Mb	*Rustaqi*	*Jenoubi*	*Rustaqi*	*Jenoubi*			
26	5017714	5578654	560940	N	Y	N	Y	*PCDH15*	Healthy related<br>Maintenance of intestine membrane. Also, it plays an essential role in maintenance of normal retinal and cochlear function.	Gene card
26	6906081	8343629	1437548	Y	Y	N	Y	*PRKG1*	Immunological and Healthy related<br>Associated with tick resistance. Milk fatty acid traits, Daughter pregnancy rate and udder structure (height, cleft, and attachment, Teat placement)	(Mapholi *et al.*, 2016)<br>(Vajana *et al.*, 2018)<br>QTL<br>(Li *et al.*, 2014)<br>(Gautier *et al.*, 2006)
26	20694707	20777487	82780	Y	N	Y	N	*DNMBP*	Healthy related<br>Milk-fat composition trait	(Buitenhuis *et al.*, 2014)
26	20206332	20276715	70383	Y	N	N	N	*CNNM1*	Healthy related<br>Functions as a copper storage protein in neuronal cells	(Chandran *et al.*, 2016)
26	20494069	20524328	30259	Y	N	N	N	*ENTPD7*	Immunological and Healthy related<br>Role in hepatic purinergic signalling gene network expression and its relationship with inflammation and oxidative stress. Starch and sucrose metabolism.	(Seo *et al.*, 2014)<br>(Kim *et al.*, 2015)
26	20613538	20684065	70527	Y	N	N	N	*ABCC2*	Immunological and Healthy related<br>Play a fundamental role in resistance to gastrointestinal nematodes. Also, play role in intestinal, liver drug efflux mechanisms	(Li *et al.*, 2011)<br>(Zancanella *et al.*, 2013)

Chromosome	Candidate gene regions			*iHS* = 4 /- 4 (threshold)		*Rsb* = 5 /- 5 (threshold)		Gene ID	Trait association / QTL information (Biological role)	Studies
	Start	*End*	Mb	*Rustaqi*	*Jenoubi*	*Rustaqi*	*Jenoubi*			
26	21010622	21035035	24413	Y	N	N	N	*CWF19L1*	Healthy related<br>Skeletal muscle development and regeneration in cattle	(Moreno-Sánchez *et al.*, 2010)
26	21248525	21280208	31683	Y	N	N	N	*SEC31B*	Healthy related<br>Play role in milk fatty acid (FA) trait	(Buitenhuis *et al.*, 2014)

- **Y: significant result, N: non-significant result.**

References

Abo-Ismail, K., V ander V oort, G., Squires, J., Swanson, C., Mandell, B., Liao, X., Stothard, P ., Moore, S., Plastow, G. and Miller, P. (2014). Single nucleotide polymorphisms for feed efficiency and performance in crossbred beef cattle. *BMC Genetics*, 15(1), p.1.

Al Naib, A., Mamo, S., O'Gorman, M., Lonergan, P., Swales, A. and Fair, T. (2011). Regulation of non-classical major histocompatability complex class I mRNA expression in bovine embryos. *Journal of Reproductive Immunology*, 91(1), pp.31-40.

Allan, F., Kuehn, A., Cushman, A., Snelling, M., Echternkamp, E. and Thallman, M. (2009). Confirmation of quantitative trait loci using a low-density single nucleotide polymorphism map for twinning and ovulation rate on bovine chromosome 5. *Journal of Animal Science*, 87(1): 46-56.

Barbisin, M., Vanni, S., Schmädicke, C., Montag, J., Motzkus, D., Opitz, L., Salinas-Riester, G. and Legname, G. (2014). Gene expression profiling of brains from bovine spongiform encephalopathy (BSE)-infected cynomolgus macaques. *BMC Genomics*, 15(1),434. doi:10.1186/1471-2164-15-434.

Barthelemy, A., Sencio, V., Soulard, D., Deruyter, L., Faveeuw, C., Le Goffic, R., and Trottein, F. (2018). Interleukin-22 immunotherapy during severe influenza enhances lung tissue integrity and reduces secondary bacterial systemic invasion. *Infection and immunity*, 86(7), e00706-17 IAI. 00706-17. doi: 10.1128/IAI.00706-17.

Bradley, L., Yaworsky, J. and Walsh, S. (2008). Myostatin as a therapeutic target for musculoskeletal disease. *Cellular and Molecular Life Sciences*, 65(14),2119-2124.

Chandran, U., Indu, S., Kumar, A.T., Devi, N., Khan, I., Srivastava, D. and Kumar, G. (2016). Expression of Cnnm1 and its association with stemness, cell cycle, and differentiation in spermatogenic cells in mouse testis. *Biology of Reproduction*, 95(1):7,1–12. doi 10.1095/biolreprod.115.130369.

Cheng, Z., Oguejiofor, F., Swangchan-Uthai, T., Carr, S. and Wathes, C. (2015). Relationships between Circulating Urea Concentrations and Endometrial Function in Postpartum Dairy Cows. *Animals*, 5(3),748-773.

Do, D. N., Bissonnette, N., Lacasse, P., Miglior, F., Sargolzaei, M., Zhao, X., and Ibeagha-Awemu, E. M. (2017). Genome-wide association analysis and pathways enrichment for lactation persistency in Canadian Holstein cattle. *Journal of dairy science*, 100(3), 1955-1970. doi: 10.3168/jds.2016- 11910.

Edea, Z., Dadi, H., Dessie, T., Uzzaman, M. R., Rothschild, M. F., Kim, E. S., and Kim, K. S. (2018). Genome-wide scan reveals divergent selection among taurine and zebu cattle populations from different regions. *Animal genetics*.9(6), 550-563. doi: 10.1111/age.12724.

Finelli, J., Sanchez-Pulido, L., Liu, X., Davies, E. and Oliver, L. (2016). The evolutionarily conserved Tre2/Bub2/Cdc16 (TBC), Lysin motif (LysM), Domain catalytic (TLDc) domain is neuroprotective against oxidative stress. *Journal of Biological Chemistry*, *291*(6),2751-2763.

Flisikowski, K., Flisikowska, T., Sikorska, A., Perkowska, A., Kind, A., Schnieke, A. and Switonski, M. (2015). Germline gene polymorphisms predisposing domestic mammals to carcinogenesis. *Veterinary and Comparative Oncology*. 15(2), 289-298.DOI: 10.1111/vco.12186.

Garbe, R. (2015). *Functional Annotation of the Bovine and Porcine Genomes*. Doctoral dissertation, University of Minnesota, Minnesota -USA. Available online at: http://hdl.handle.net/11299/175436.

Gautier, M., Barcelona, R., Fritz, S., Grohs, C., Druet, T., Boichard, D., Eggen, A. and Meuwissen, H. (2006). Fine mapping and physical characterization of two linked quantitative trait loci affecting milk fat yield in dairy cattle on BTA26. *Genetics*, 172(1),425-436.

Guo, B., Greenwood, L., Cafe, M., Zhou, G., Zhang, W. and Dalrymple, P. (2015). Transcriptome analysis of cattle muscle identifies potential markers for skeletal muscle growth rate and major cell types. *BMC Genomics,* 16(1), 177. doi:10.1186/s12864-015-1403-x.

Hallé, C., Goff, K., Petit, V., Blouin, R. and Palin, F. (2015). Effects of different n-6: n-3 fatty acid ratios and of enterolactone on gene expression and PG secretion in bovine endometrial cells. *British Journal of Nutrition, 113*(01),56-71.

Hendriks, J., Dilaver, G., Noordman, E., Kremer, B. and Fransen, A. (2009). PTPRR protein tyrosine phosphatase isoforms and locomotion of vesicles and mice. *The Cerebellum, 8*(2),80-88.

Jeong, H., Song, D., Seo, M., Caetano-Anollés, K., Kim, J., Kwak, W., Oh, D., Kim, E., Jeong, K., Cho, S. and Kim, H. (2015). Exploring evidence of positive selection reveals genetic basis of meat quality traits in Berkshire pigs through whole genome sequencing. *BMC Genetics,* 16(1), 104. doi:10.1186/s12863-015-0265-1.

Kärst, S., Cheng, R., Schmitt, O., Yang, H., de Villena, M., Palmer, A. and Brockmann, A. (2011). Genetic determinants for intramuscular fat content and water-holding capacity in mice selected for high muscle mass. *Mammalian Genome, 22*(9-10),530-543.

Khatri, P., Frenette, G., Sullivan, R., Hoffmann, B. and Schuler, G. (2011). Expression of SULT1E1 protein in bovine placentomes: evidence for localization in uninucleated trophoblast cells. *Placenta, 32*(6),431-440.

Mamiya, C., Hennesy, Z., Zhou, R. and Wagner, C. (2008). Changes in attack behavior and activity in EphA5 knockout mice. *Brain Rresearch*, 1205, pp.91-99.

Martchenko, D., Prewer, E., Latch, E. K., Kyle, C. J., and Shafer, A. B. (2018). *Population Genomics of Ungulates*. Springer International Publishing, Canada. doi: org/10.1007/13836_2018_30.

McCabe, M., Waters, S., Morris, D., Kenny, D., Lynn, D. and Creevey, C. (2012). RNA-seq analysis of differential gene expression in liver from lactating dairy cows divergent in negative energy balance. *BMC Genomics*, 13(1), 193. doi:10.1186/1471-2164-13-193

Mészáros, G., Eaglen, S. and Waldmann, P. (2014). A genome wide association study for longevity in cattle. *Open Journal of Genetics*. 4, (1) 46. doi: 10.4236/ojgen.2014.41007

Moreno-Sánchez, N., Rueda, J., Carabaño, J., Reverter, A., McWilliam, S., González, C. and Díaz, C. (2010). Skeletal muscle specific genes networks in cattle. *Functional and Integrative Genomics*, 10(4),609-618.

Ortega, S., Denicol, C., Cole, B., Null, J. and Hansen, J. (2016). Use of single nucleotide polymorphisms in candidate genes associated with daughter pregnancy rate for prediction of genetic merit for reproduction in Holstein cows. *Animal Genetics*. *47*, 288–297, doi: 10.1111/age.12420.

Pandey, K., Mizukami, Y., Watanabe, K., Sakaguti, S., and Kadokawa, H. (2017). Deep sequencing of the transcriptome in the anterior pituitary of heifers before and after ovulation. *Journal of Veterinary Medical Science*, 79(6), 1003-1012. doi: 10.1292/jvms.16-0531

Pausch, H., Wurmser, C., Edel, C., Emmerling, R., Götz, U. and Ruedi, F. (2014). Exploiting whole genome sequence data for the identification of causal trait variants in cattle. *Proc. 10th World Congress. Genetics Applied Livestock Production. Vancouver, Canada: WCGALP*. doi:10.13140 /2.1.1397.6 966

Perdigao, F., Gomez, S., Pimenta, S. and De Marco, L. (2004). Ameloblastin gene (AMBN) mutations associated with epithelial odontogenic tumors. *Oral Oncology*, *40*(8), 841-846.

Ponsuksili, S., Murani, E., Schwerin, M., Schellander, K., Tesfaye, D. and Wimmers, K. (2012). Gene expression and DNA-methylation of bovine pretransfer endometrium depending on its receptivity after in vitro-produced embryo transfer. *PloS One*, *7*(8), p.e42402.

Qanbari, S., Pimentel, G., Tetens, J., Thaller, G., Lichtner, P., Sharifi, R. and Simianer, H. (2010). A genome-wide scan for signatures of recent selection in Holstein cattle. *Animal Genetics,* 41(4), 377-389. doi:10.1111/j.1365-2052.2009. 02016.x

Randhawa, A., Khatkar, S., Thomson, C. and Raadsma, W. (2016). A Meta-Assembly of Selection Signatures in Cattle. *PloS One,* 11(4), p. e0153013. doi: 10.1371/journal.pone.0153013

Ren, S., Lu, G., Ota, A., Zhou, H., Vondriska, M., Lane, F. and Wang, Y. (2014). IRE1 phosphatase PP2Ce regulates adaptive ER stress response in the postpartum mammary gland. *PloS One.* 9(11), p.e111606. doi: 10.1371/journal.pone.0111606

Ringseis, R., Windisch, W. and Eder, K. (2016). Transcript profiling in the liver of early-lactating dairy cows fed conjugated linoleic acid. *Genomics Data,* 10, 101-103. doi:10.1016/.gdata. 2016.10.004

Rothammer, S., Seichter, D., Förster, M., and Medugorac, I. (2013). A genome-wide scan for signatures of differential artificial selection in ten cattle breeds. *BMC Genomics,* 14(1), 908. doi:10.1186/1471-2164-14-908

Seo, J., Osorio, S., Schmitt, E., Corrêa, N., Bertoni, G., Trevisi, O. and Loor, J. (2014). Hepatic purinergic signaling gene network expression and its relationship with inflammation and oxidative stress biomarkers in blood from peripartal dairy cattle. *Journal of Dairy Science, 97*(2),861-873. doi:10.3168/jds.2013-7379

Seong, J., Yoon, H. and Kong, S. (2016). Identification of microRNA and target gene associated with marbling score in Korean cattle (Hanwoo). *Genes and Genomics,* 38(6),529-538. doi :10.100 7/s13258-016-0401-y

Sevane, N., Armstrong, E., Wiener, P., Wong, P., Dunner, S. and GemQual Consortium, (2014). Polymorphisms in twelve candidate genes are associated with growth, muscle lipid profile and meat quality traits in eleven European cattle breeds. *Molecular Biology Reports,* 41(7), 4721-4731. doi.org/10.1007/s11033-014-3343-y

Shin, H., Lee, J., Cho, S., Kim, J., Hwang, Y., Lee, K., Jeong, J., Yoon, D. and Kim, H. (2014). Deleted copy number variation of Hanwoo and Holstein using next generation sequencing at the population level. *BMC Genomics*, 15(1),240. doi:10.1186/1471-2164-15-240

Stefely, A., Licitra, F., Laredj, L., Reidenbach, G., Kemmerer, A., Grangeray, A., Jaeg-Ehret, T., Minogue, E., Ulbrich, A., Hutchins, D. and Wilkerson, M. (2016). Cerebellar ataxia and coenzyme Q deficiency through loss of unorthodox kinase activity. *Molecular Cell. 63*(4), 608- 620.

Ushizawa, K., Takahashi, T., Hosoe, M., Ishiwata, H., Kaneyama, K., Kizaki, K. and Hashizume, K. (2007). Global gene expression analysis and regulation of the principal genes expressed in bovine placenta in relation to the transcription factor AP-2 family. *Reproductive Biology and Endocrinology, 5*(1),17. doi:10.1186/1477-7827-5-17

Walser, M. (2012). *Validation of P2RX7 and TMEM132D as susceptibility genes for depression using genetic mouse models*. Doctoral dissertation, München, Technische Universität München, Diss, Germany. Available online at: https://mediatum.ub.tum.de/doc/1093008/1093008.pdf

Wang, Q., Xing, M., Fan, H., Wang, S., Zhang, K., Wang, W., Qi, J., Yang, M., Yang, J., Ren, N. and Cui, J. (2009). The novel lipopolysaccharide-binding protein CRISPLD2 is a critical serum protein to regulate endotoxin function. *The Journal of Immunology*, 183(10),6646-6656. doi:10.4049/jimmunol.0802348

Wu, J., Zhang, J., Teng, M., Yang, L., Yi, J., Ha, G., Cooke, S., Clempson, M., Pollot, E., Wathes, C. and Zhang, S. (2014). Genome-wide association study of fertility traits in dairy cows. *Advances in Animal Biosciences, 5*(2),256. doi:10.1017/S2040470014000090

Yu, L., Lee, M., Kang, J., Jeong, J., Sang, C., Jeon, T. and Lee, H. (2009). Identification of differentially expressed genes between preadipocytes and adipocytes using affymetrix bovine genome array. *Journal of Animal Science and Technology*, 51(6),443-452. doi: 10.5187/JA ST.2009.51.6.443

Zancanella, V., Giantin, M., Lopparelli, M., Nebbia, C. and Dacasto, M. (2013). Tissue distribution and phenobarbital induction of target SLC-and ABC-transporters in cattle. *Journal of Veterinary Pharmacology and Therapeutics*, 36(4),358-369. doi.org/10.1111/j.1365-2885. 2012.01427.x

Zhang, Z. (2012). *Influence of Dietary Selenium Supplementation Form on Hepatic Transcriptome Profiles of Maturing Beef Heifers*.75-76. Master thesis, College of Agriculture, University of Kentucky, USA. Available online at: https://uknowledge.uky.edu/cgi/viewcontent.cgi?referer=&httpsredir=1&article=1006&context=animalsci_etds
