# Supplementary material for: Genome Analysis Reveals Genetic Admixture and Signature of Selection for Productivity and Environmental Traits in Iraqi Cattle
Source: Front Genet. 2019 Jul 16;10:609. doi: 10.3389/fgene.2019.00609 (PMC6646475; doi:10.3389/fgene.2019.00609)
Supplement: Supplementary file 7 [file Table_7.pdf]

Supplementary Table S7: Candidate regions with their SNP positions detected by *iHS* and *Rsb* in Iraqi breeds (Rustaqi and Jenoubi) (Red colour refer to genes associated with the innate or acquired immune response)

**Chr.No.**	**Candidate gene**		***iHS* = 4 /- 4 (threshold)**		***Rsb* = 5 /- 5 (threshold)**		**Gene ID**	**SNPs positions of this region (bp)**	Log (*P*-value) of highest SNP within each region
	**Start**	**End**	**Rustaqi**	**Jenoubi**	**Rustaqi**	**Jenoubi**			
1	56722581	56818961	N	N	Y	N	*CD96-201*	56794098, 56798699	8.24
1	14791090	15026555	N	Y	N	Y	*NCAM2*	14807932, 14811112, 14814356, 14830358, 14836256, 14845369, 14856702, 14862398, 14883822, 14894352, 14901598, 14909272, 14912192, 14912759, 14931292, 14936349, 14941571,14947461, 14961305, 14961941	12.95
1	18058709	18207251	N	Y	N	Y	*TMPRSS15*	18068017, 18100363, 18106896, 18110707, 18124439, 18127125, 18131757, 18136336, 18137402, 18142738, 18145688, 18150638, 18156795, 18165990, 18168044, 18169642, 18170722, 18171811, 18172453, 18173093, 18172451, 18173944, 18182821, 18186366, 18188932, 18195508, 18203388,	12.93

1	18208866	18233154	N	N	N	Y	*CHODL*	18211250, 18214381, 18220551, 18224393, 18225013, 18228423, 18231821	12.92
1	107241227	107576086	Y	N	N	N	*PPM1L*	107535813, 107544870, 107545399, 107552580, 107555692, 107556936	7.68
1	141349995	141401851	Y	N	N	N	*IGSF5*	141397128	4.21
3	103353700	103422206	Y	N	N	N	*CFAP57*	103364954	4.37
5	43746464	43872342	N	N	Y	N	*MYRFL-201*	43763706, 43765475, 43766372, 43778019, 43790134	7.90
5	33189704	33339728	Y	N	N	N	*PCED1B*	33301009	4.35
5	42837597	43112731	Y	N	N	N	*PTPRR*	43019261	4.26
5	91835146	92276939	Y	N	N	N	*PIK3C2G*	92162494, 92167965	4.93
6	82560093	82962887	N	Y	N	N	*EPHA5*	82898792, 82905087,82912559	4.29
6	88182303	88541046	N	Y	N	Y	*SLC4A4*	88226031	4.33
6	88695940	88739180	N	Y	N	Y	*GC*	88711884, 88704865, 88711884, 88720585, 88723381, 88724389, 88728581	4.80 (*iHS*), 6.69 (*Rsb*)
6	90842934	90985937	N	Y	N	N	*MTHFD2L*	90913463	4.42
6	86169557	86190786	N	N	N	Y	*Novel*	86169865	5.06
6	87035926	87094952	N	N	N	Y	*SULT1E1*	87066479	7.13
6	87179502	87188025	N	N	N	Y	*CSN2*	87186813	7.34

6	87694412	87705727	N	N	N	Y	*AMBN*	87700768, 87705515	6.92
6	89162542	89460195	N	N	N	Y	*ADAMTS3*	89243676, 89419440, 89432775, 89439849, 89440731, 89446025, 89450857, 89458909	7.17
6	91597122	91723390	N	N	N	Y	*PARM1*	91625207, 91627211, 91628097, 91634816, 91636147, 91641948, 91656338	7.76
7	71003540	71115619	N	N	N	Y	*CYFIP2*	71040064	6.42
7	73928475	73960160	N	N	N	Y	*CCNJL*	73928988, 73930656, 73943295	7.22
7	35812577	35858617	N	Y	N	N	*TNFAIP8*	35818176	4.62
7	69585066	70027839	N	Y	N	N	*SGCD*	69830107	4.21
7	12206055	12266930	Y	N	N	N	*ADGRE3*	12234576	4.35
8	23386997	23662561	N	Y	N	Y	*FOCAD*	23654320, 23661626, 23443615	7.33
10	44711724	44842190	Y	N	N	N	*GNG2*	44821159	4.50
10	48949618	49750993	Y	N	N	N	*RORA* *(RORA)*	49350661	4.42
10	51020033	51240077	Y	N	N	N	*MYO1E*	51131338, 51136557, 51137424	4.82
11	38706659	38928773	Y	N	N	N	*CCDC85A*	38859018	5.25
13	36610252	36833259	Y	N	N	N	*MPP7*	36623846	4.20
13	39202450	39632869	Y	N	N	N	*SLC24A3*	39429040, 39444606	5.10
13	43605579	43610260	Y	N	N	N	*UCN3*	43602483, 43606026	4.09

16	30656001	30703396	Y	N	N	N	*COQ8A*	30682132	4.49
16	32381118	32471604	Y	N	N	N	*KIF26B*	32436360	4.24
16	45621457	45879645	Y	N	N	N	*RERE*	45871503	4.22
17	52565556	52785519	N	N	N	Y	*TMEM132B*	52740162, 52742056, 52744905	6.54
18	8427455	8516798	Y	N	N	N	*PLCG2*	8509101	4.90
18	9512739	10162782	Y	N	N	N	*CDH13*	9522978, 9577756, 9613861, 9621108, 9577756, 9613861, 9621108, 9621999, 9627742, 9855020, 9931181, 9995816, 10072719, 10158499	7.55
18	10250180	10263073	Y	N	N	N	*NOVEL*	10253121	5.58
18	10294996	10305608	Y	N	N	N	*OSGIN1*	10300647	5.95
18	10694237	10706275	Y	N	N	N	*TLDC1*	10701992, 10704759, 10706032	6.33
18	10985132	11050904	Y	N	N	N	*CRISPLD2*	10994466, 11031658, 11049213	5.309
18	11883376	11905043	Y	N	N	N	*IRF8*	11903542	4.43
18	13186023	13260798	Y	N	N	N	*JPH3*	13194141, 13197861, 13219476, 13226954, 13227501,13232056	5.71
18	13268589	13299788	Y	N	N	N	*KLHDC4*	13271751,13298075	4.33
18	13341371	13370494	Y	N	N	N	*SLC7A5*	13353611	4.40
18	13389828	13412237	Y	N	N	N	*CA5A*	13404310	4.75
18	13425303	13493366	Y	N	N	N	*BANP*	13430591,13436491, 13442089	4.27

18	14061951	14077096	Y	N	N	N	*GALNS*	14065359, 14067845,14072919	4.68
18	14096667	14174040	Y	N	N	N	*CBFA2T3*	14100421, 14106949, 14167943	4.46
18	16551061	16610345	Y	N	N	N	*ABCC12*	16600201	4.52
18	18041277	18392207	Y	N	N	N	*ZNF423*	18265206,18266068	4.24
18	23861829	23927174	Y	N	N	N	*LPCAT2*	23870174	4.91
18	39221305	39300130	Y	N	N	N	*PKD1L3*	39235401	4.33
21	35415163	35655955	N	Y	N	N	*STXBP6*	35490800, 35491930, 35624315, 35634291, 35636045	4.48
22	55944219	56217921	N	Y	N	N	*ATG7*	56108702	4.21
22	56530332	56533155	N	N	N	Y	*TRH*	56531951	6.46
24	33386429	33419545	Y	N	N	N	*ANKRD29*	33398641	5.16
26	5017714	5578654	N	Y	N	Y	*PCDH15*	5039333, 5040354, 5046750, 5049038, 5050912, 5056600, 5057838, 5067581, 5071284,5092128, 5094776, 5096038, 5102794, 5129234, 5133515, 5136452, 5137440, 5142001, 5144319, 5156224, 5158043, 5150315, 5151813, 5153748, 5154973, 5176968,5182239, 5186735, 5188315, 5189531, 5239940, 5272489, 5286405, 5288263, 5296396,5308844, 5310546, 5315170, 5317018,5429989, 5432356, 5453723, 5455336,5434687, 5449700, 5460865, 5461749, 5462770,	5.15 (*iHS*), 13.01 (*Rsb*)

								5478726, 5483737, 5486210, 5486971, 5490556, 5501248, 5518552, 5541052, 5557394, 5572185	
26	6906081	8343629	Y	Y	N	Y	*PRKG1*	6965913,6970122,7026224,7027919,7120756, 7211242,7223662,7224265,7225242,7235774,7239591,7240987,7242174,7245383,7248541,7249270,7639670,7665611,8016399,8032364,8054175,8061101,8063089,8086603,8087381	5.92(*iHS*), 7.93(*Rsb*)
26	20694707	20777487	Y	N	Y	N	*DNMBP*	20714265, 20716721, 20741742, 20745437, 20747509, 20754192, 20755727,20761561, 20757194	8.01
26	20206332	20276715	Y	N	N	N	*CNNM1*	20259486	4.88
26	20494069	20524328	Y	N	N	N	*ENTPD7*	20498173,20499135, 20506934, 20519563	5.64
26	20613538	20684065	Y	N	N	N	*ABCC2*	20614545, 20621574, 20625069, 20643699 20651745	5.39
26	21010622	21035035	Y	N	N	N	*CWF19L1*	21025549	4.21
26	21248525	21280208	Y	N	N	N	*SEC31B*	21278993	4.37

- **Y: significant result, N: non-significant result.**
- **A total of 69 genes including 1 gene found in Jenoubi and Rustaqi are present within significant regions. The common Jenoubi and Rustaqi gene is *PRKG1* in chromosome 26.**
