# Supplementary material for: Genome Analysis Reveals Genetic Admixture and Signature of Selection for Productivity and Environmental Traits in Iraqi Cattle
Source: Front Genet. 2019 Jul 16;10:609. doi: 10.3389/fgene.2019.00609 (PMC6646475; doi:10.3389/fgene.2019.00609)
Supplement: Supplementary file 8 [file Table_8.pdf]

Supplementary Table S8: Overlap and non-overlap candidate genes detected by analysis of *iHS* and *Rsb* (Rustaqi and Jenoubi) with *Rsb* analysis of Jenoubi *versus* Holstein, Nellore and N'Dama Guinea breeds.

Chr. Name	Candidate gene		*iHS* = 4 /- 4 (threshold)		*Rsb* = 5 /- 5 (threshold)		*Rsb* = 3.5 threshold	*Rsb* = 4 threshold	*Rsb* = 4 threshold	Gene ID
	Start	End	Rustaqi	Jenoubi	Rustaqi	Jenoubi	Jenoubi X Holstein	Jenoubi X Nellore	Jenoubi X N'Dama G.	
1	43730028	44124279	N	N	N	N	Y	N	N	*CMSS1*
1	56722581	56818961	N	N	Y	N	N	N	N	*CD96-201*
1	14791090	15026555	N	Y	N	Y	N	N	Y	*NCAM2*
1	18058709	18207251	N	Y	N	Y	N	N	Y	*TMPRSS15*
1	18208866	18233154	N	N	N	Y	N	N	Y	*CHODL*
1	107241227	107576086	Y	N	N	N	N	N	N	*PPM1L*
1	141349995	141401851	Y	N	N	N	N	N	N	*IGSF5*
3	103353700	103422206	Y	N	N	N	N	N	N	*CFAP57*
4	119522032	119565916	N	N	N	N	Y	N	N	*Novel*
4	120133801	120207828	N	N	N	N	Y	N	N	*NCAPG2*
4	120230771	120306448	N	N	N	N	Y	N	N	*ESYT2*
4	120326813	120373331	N	N	N	N	Y	N	N	*WDR60*
4	120427915	120494453	N	N	N	N	Y	N	N	*VIPR2*
5	121181282	121188623	N	N	N	N	N	N	Y	*TRABD*
5	43746464	43872342	N	N	Y	N	N	N	N	*MYRFL-201*
5	47534237	47703486	N	N	N	N	Y	N	N	*GRIP1*
5	47713520	47751430	N	N	N	N	Y	N	N	*HELB*
5	109152548	109417890	N	N	N	N	Y	N	N	*CACNA1C*
5	109564503	109578159	N	N	N	N	Y	N	N	*ATP6V1E1*
5	109584777	109635907	N	N	N	N	Y	N	N	*Novel*
5	109664103	109737010	N	N	N	N	Y	N	N	*MICAL3*
5	117677522	117738845	N	N	N	N	Y	N	N	*GTSE1*
5	117743264	117758670	N	N	N	N	Y	N	N	*TRMU*
5	117764821	117853214	N	N	N	N	Y	N	N	*Novel*
5	120933369	120940333	N	N	N	N	Y	N	N	*CRELD2*
5	121012667	121067541	N	N	N	N	Y	N	N	*TTLL8*
5	121099143	121164873	N	N	N	N	Y	N	Y	*MOV10L1*

5	33189704	33339728	Y	N	N	N	N	N	N	*PCED1B*
5	42837597	43112731	Y	N	N	N	N	N	N	*PTPRR*
5	91835146	92276939	Y	N	N	N	N	N	N	*PIK3C2G*
6	82560093	82962887	N	Y	N	N	N	N	N	*EPHA5*
6	88182303	88541046	N	Y	N	Y	N	N	N	*SLC4A4*
6	88695940	88739180	N	Y	N	Y	N	N	N	*GC*
6	90842934	90985937	N	Y	N	N	N	N	N	*MTHFD2L*
6	91126256	91138391	N	N	N	N	N	N	Y	*AREG*
6	86169557	86190786	N	N	N	Y	N	N	N	*Novel*
6	87035926	87094952	N	N	N	Y	N	N	N	*SULT1E1*
6	87179502	87188025	N	N	N	Y	N	N	N	*CSN2*
6	87694412	87705727	N	N	N	Y	N	N	N	*AMBN*
6	89162542	89460195	N	N	N	Y	N	N	N	*ADAMTS3*
6	91597122	91723390	N	N	N	Y	N	N	Y	*PARM1*
7	71003540	71115619	N	N	N	Y	N	N	Y	*CYFIP2*
7	73928475	73960160	N	N	N	Y	N	N	N	*CCNJL*
7	35812577	35858617	N	Y	N	N	N	N	N	*TNFAIP8*
7	69585066	70027839	N	Y	N	N	N	N	N	*SGCD*
7	12206055	12266930	Y	N	N	N	N	N	N	*ADGRE3*
8	30529	228065	N	N	N	N	Y	N	N	*HIATL1*
8	23386997	23662561	N	Y	N	Y	N	N	Y	*FOCAD*
9	104927415	105066196	N	N	N	N	Y	N	N	*WDR27*
10	44711724	44842190	Y	N	N	N	N	N	N	*GNG2*
10	48949618	49750993	Y	N	N	N	N	N	N	*RORA (RORA)*
10	47303876	47796327	N	N	N	N	Y	N	N	*TLN2*
10	51020033	51240077	Y	N	N	N	N	N	N	*MYO1E*
11	38706659	38928773	Y	N	N	N	N	N	N	*CCDC85A*
11	61759950	61969748	N	N	N	N	Y	N	N	*WDPCP*
11	101350157	101438043	N	N	N	N	Y	N	N	*NUP214*
12	27866074	28033238	N	N	N	N	Y	N	N	*STARD13*
13	36610252	36833259	Y	N	N	N	N	N	N	*MPP7*
13	39202450	39632869	Y	N	N	N	N	N	N	*SLC24A3*

13	43605579	43610260	Y	N	N	N	N	N	N	*UCN3*
15	30023047	30033583	N	N	N	N	Y	N	N	*BCL9L*
16	30656001	30703396	Y	N	N	N	N	N	N	*COQ8A*
16	32381118	32471604	Y	N	N	N	N	N	N	*KIF26B*
16	45621457	45879645	Y	N	N	N	N	N	N	*RERE*
17	52565556	52785519	N	N	N	Y	Y	N	N	*TMEM132B*
18	8427455	8516798	Y	N	N	N	N	N	N	*PLCG2*
18	9512739	10162782	Y	N	N	N	N	N	N	*CDH13*
18	10250180	10263073	Y	N	N	N	N	N	N	*NOVEL*
18	10294996	10305608	Y	N	N	N	N	N	N	*OSGIN1*
18	10694237	10706275	Y	N	N	N	N	N	N	*TLDC1*
18	10985132	11050904	Y	N	N	N	N	N	N	*CRISPLD2*
18	11883376	11905043	Y	N	N	N	N	N	N	*IRF8*
18	13186023	13260798	Y	N	N	N	N	N	N	*JPH3*
18	13268589	13299788	Y	N	N	N	N	N	N	*KLHDC4*
18	13341371	13370494	Y	N	N	N	N	N	N	*SLC7A5*
18	13389828	13412237	Y	N	N	N	N	N	N	*CA5A*
18	13425303	13493366	Y	N	N	N	N	N	N	*BANP*
18	14061951	14077096	Y	N	N	N	N	N	N	*GALNS*
18	14096667	14174040	Y	N	N	N	N	N	N	*CBFA2T3*
18	16551061	16610345	Y	N	N	N	N	N	N	*ABCC12*
18	18041277	18392207	Y	N	N	N	N	N	N	*ZNF423*
18	23861829	23927174	Y	N	N	N	N	N	N	*LPCAT2*
18	39221305	39300130	Y	N	N	N	N	N	N	*PKD1L3*
21	35415163	35655955	N	Y	N	N	N	N	N	*STXBP6*
22	55944219	56217921	N	Y	N	N	Y	N	N	*ATG7*
22	56530332	56533155	N	N	N	Y	N	N	N	*TRH*
22	59460406	59549010	N	N	N	N	Y	N	N	*IQSEC1*
24	582652	659851	N	N	N	N	Y	N	N	*PARD6G*
24	680619	701258	N	N	N	N	Y	N	N	*ADNP2*
24	729251	741978	N	N	N	N	Y	N	N	*RBFA*
24	743787	776146	N	N	N	N	Y	N	N	*NOVEL*
24	33386429	33419545	Y	N	N	N	N	N	N	*ANKRD29*

26	6344781	6348912	N	N	N	N	N	N	Y	*MBL2*
26	5017714	5578654	N	Y	N	Y	Y	Y	Y	***PCDH15***
26	6906081	8343629	Y	Y	N	Y	Y	N	Y	*PRKG1*
26	20694707	20777487	Y	N	Y	N	N	N	N	*DNMBP*
26	20206332	20276715	Y	N	N	N	N	N	N	*CNNM1*
26	20494069	20524328	Y	N	N	N	N	N	N	*ENTPD7*
26	20613538	20684065	Y	N	N	N	N	N	N	*ABCC2*
26	21010622	21035035	Y	N	N	N	N	N	N	*CWF19L1*
27	28963365	28969954	N	N	N	N	Y	N	N	*DUSP26*
28	25073149	25191276	N	N	N	N	Y	N	Y	*TET1*
28	44545580	44597645	N	N	N	N	Y	N	N	*ZFAND4*
28	44545580	44597645	N	N	N	N	Y	N	N	*NOVEL*
28	44612704	44722610	N	N	N	N	Y	N	N	*NOVEL*

- Y indicate the test detect the gene region, N indicate the test did not detect the gene region.
- Red colour lines refer to immunity genes.
- Rsb for Jenoubi X Holstein, revealed 38 genes in Jenoubi (**green colour**): 4 genes shared between Jenoubi here and previous genes that appeared in just Iraqi analysis. These genes are ATG7 and PRKG1 (immunity genes) and PCDH15 and TMEM132B. While, remained 34 genes consider as new genes not appeared in our previous analysis regarding Iraqi breeds.
- Only one gene appeared in *Rsb* Jenoubi X Nellore analysis. This gene is PCDH15 (**violet colour**) and it appeared also in our previous Iraqi analysis.
- In Rsb Jenoubi X N'Dama G., 13 genes appeared (**brown colour**), 8 genes appeared in Iraqi breeds analysis (NCAM2, TMPRSS15, CHODL, PCDH15, PRKG1, PARM1, CYFIP2, FOCAD). Moreover, 1 gene appeared in Rsb Jenoubi X Holstein Analysis (TET1).
