# Supplementary material for: Genome Analysis Reveals Genetic Admixture and Signature of Selection for Productivity and Environmental Traits in Iraqi Cattle
Source: Front Genet. 2019 Jul 16;10:609. doi: 10.3389/fgene.2019.00609 (PMC6646475; doi:10.3389/fgene.2019.00609)
Supplement: Supplementary file 9 [file Table_9.pdf]

Supplementary Table S9: Overlap and non-overlap candidate genes detected by analysis of *iHS* and *Rsb* (Rustaqi and Jenoubi) with *Rsb* analysis of Rustaqi *versus* Holstein, Nellore and N'Dama Guinea breeds.

Chr. Name	Candidate gene		*iHS* = 4 /- 4 (threshold)		*Rsb* = 5 /- 5 (threshold)		*Rsb* = 3.5 threshold	*Rsb* =3 threshold	*Rsb* = 3.5 threshold	Gene ID
	Start	End	Rustaqi	Jenoubi	Rustaqi	Jenoubi	Rustaqi X Hol.	Rustaqi X Nellore	Rustaqi X N' Dama G.	
1	43730028	44124279	N	N	N	N	Y	N	N	*CMSS1*
1	56722581	56818961	N	N	Y	N	N	N	Y	*CD96-201*
1	14791090	15026555	N	Y	N	Y	N	N	N	*NCAM2*
1	18058709	18207251	N	Y	N	Y	N	N	N	*TMPRSS15*
1	18208866	18233154	N	N	N	Y	N	N	N	*CHODL*
1	107241227	107576086	Y	N	N	N	N	N	N	*PPM1L*
1	112455492	112013084	N	N	N	N	N	Y	N	*KCNAB1*
1	141349995	141401851	Y	N	N	N	N	N	N	*IGSF5*
3	8985751	8969737	N	N	N	N	N	N	Y	*SLAMF7*
3	8985751	8969737	N	N	N	N	N	N	Y	*SLAMF6*
3	103353700	103422206	Y	N	N	N	N	N	N	*CFAP57*
4	46912545	46858942	N	N	N	N	N	N	Y	*PUS7*
4	47228658	46974646	N	N	N	N	N	N	Y	*ATXN7L1*
4	117411239	117153120	N	N	N	N	N	Y	N	*Novel*
4	119522032	119565916	N	N	N	N	Y	N	N	*Novel*
4	120133801	120207828	N	N	N	N	Y	N	N	*NCAPG2*
4	120230771	120306448	N	N	N	N	Y	N	N	*ESYT2*
4	120326813	120373331	N	N	N	N	Y	N	N	*WDR60*
4	120427915	120494453	N	N	N	N	Y	N	N	*VIPR2*
5	121181282	121188623	N	N	N	N	N	N	N	*TRABD*
5	121164873	121099143	N	N	N	N	N	N	Y	*MOV10L1*
5	121188623	121181282	N	N	N	N	N	N	Y	*TRABD*
5	43746464	43872342	N	N	Y	N	N	N	N	*MYRFL-201*
5	47534237	47703486	N	N	N	N	Y	N	N	*GRIP1*
5	47713520	47751430	N	N	N	N	Y	N	N	*HELB*

5	109152548	109417890	N	N	N	N	N	N	N	*CACNA1C*
5	109564503	109578159	N	N	N	N	N	N	N	*ATP6V1E1*
5	109584777	109635907	N	N	N	N	N	N	N	*Novel*
5	109664103	109737010	N	N	N	N	N	N	N	*MICAL3*
5	117677522	117738845	N	N	N	N	N	N	N	*GTSE1*
5	112916436	112943925	N	N	N	N	Y	N	N	*RANGAP1*
5	112977785	113010230	N	N	N	N	Y	N	N	*ZC3H7B*
5	117743264	117758670	N	N	N	N	N	N	N	*TRMU*
5	117764821	117853214	N	N	N	N	N	N	N	*Novel*
5	120933369	120940333	N	N	N	N	N	N	N	*CRELD2*
5	121012667	121067541	N	N	N	N	N	N	N	*TTLL8*
5	121099143	121164873	N	N	N	N	N	N	N	*MOV10L1*
5	33189704	33339728	Y	N	N	N	N	N	N	*PCED1B*
5	42837597	43112731	Y	N	N	N	N	N	N	*PTPRR*
5	91835146	92276939	Y	N	N	N	N	N	N	*PIK3C2G*
6	37609959	37479804	N	N	N	N	N	N	Y	*HERC3*
6	82560093	82962887	N	Y	N	N	N	N	N	*EPHA5*
6	88182303	88541046	N	Y	N	Y	N	N	N	*SLC4A4*
6	88695940	88739180	N	Y	N	Y	N	N	N	*GC*
6	90842934	90985937	N	Y	N	N	N	N	N	*MTHFD2L*
6	91126256	91138391	N	N	N	N	N	N	N	*AREG*
6	86169557	86190786	N	N	N	Y	N	N	N	*Novel*
6	87035926	87094952	N	N	N	Y	N	N	N	*SULT1E1*
6	87179502	87188025	N	N	N	Y	N	N	N	*CSN2*
6	87694412	87705727	N	N	N	Y	N	N	N	*AMBN*
6	89162542	89460195	N	N	N	Y	N	N	N	*ADAMTS3*
6	91597122	91723390	N	N	N	Y	N	N	N	*PARM1*
7	28395380	28238850	N	N	N	N	N	N	Y	*MARCH3*
7	71003540	71115619	N	N	N	Y	N	N	N	*CYFIP2*
7	73928475	73960160	N	N	N	Y	N	N	N	*CCNJL*
7	35812577	35858617	N	Y	N	N	N	N	N	*TNFAIP8*
7	69585066	70027839	N	Y	N	N	N	N	N	*SGCD*
7	12206055	12266930	Y	N	N	N	N	N	N	*ADGRE3*

8	30529	228065	N	N	N	N	Y	N	N	*HIATL1*
8	52712391	52292117	N	N	N	N	N	Y	N	*PCSK5*
8	23386997	23662561	N	Y	N	Y	N	N	N	*FOCAD*
8	92674692	92346242	N	N	N	N	N	Y	N	*PLPPR1*
8	104995986	104797238	N	N	N	N	N	N	Y	-
9	104927415	105066196	N	N	N	N	N	N	N	*WDR27*
10	21573888	21521555	N	N	N	N	N	N	Y	*SLC7A8*
10	44711724	44842190	Y	N	N	N	N	N	N	*GNG2*
10	48949618	49750993	Y	N	N	N	N	N	N	*RORA (RORA)*
10	47303876	47796327	N	N	N	N	N	N	N	*TLN2*
10	51020033	51240077	Y	N	N	N	N	N	N	*MYO1E*
11	38706659	38928773	Y	N	N	N	N	N	N	*CCDC85A*
11	61759950	61969748	N	N	N	N	N	N	N	*WDPCP*
11	64915358	64770131	N	N	N	N	N	N	Y	*MEIS1*
11	101350157	101438043	N	N	N	N	N	N	N	*NUP214*
12	27866074	28033238	N	N	N	N	N	N	N	*STARD13*
12	28110415	28073218	N	N	N	N	N	N	Y	*KL*
12	54777914	54825844	N	N	N	N	Y	N	N	*RBM26*
13	36610252	36833259	Y	N	N	N	N	N	N	*MPP7*
13	39202450	39632869	Y	N	N	N	N	N	N	*SLC24A3*
13	43605579	43610260	Y	N	N	N	N	N	N	*UCN3*
14	70303427	70236070	N	N	N	N	N	N	Y	*PTDSS1*
15	30023047	30033583	N	N	N	N	N	N	N	*BCL9L*
15	78254804	78044073	N	N	N	N	N	N	Y	*C11orf49*
16	30656001	30703396	Y	N	N	N	N	N	N	*COQ8A*
16	32381118	32471604	Y	N	N	N	N	N	N	*KIF26B*
16	45621457	45879645	Y	N	N	N	N	N	N	*RERE*
16	48314477	48181539	N	N	N	N	N	N	Y	*NPHP4*
17	52565556	52785519	N	N	N	Y	N	N	N	*TMEM132B*
18	8427455	8516798	Y	N	N	N	N	N	N	*PLCG2*

18	9512739	10162782	Y	N	N	N	N	N	N	*CDH13*
18	10250180	10263073	Y	N	N	N	N	N	N	*NOVEL*
18	10294996	10305608	Y	N	N	N	N	N	N	*OSGIN1*
18	10694237	10706275	Y	N	N	N	N	N	N	*TLDC1*
18	10985132	11050904	Y	N	N	N	N	N	N	*CRISPLD2*
18	11883376	11905043	Y	N	N	N	N	N	N	*IRF8*
18	13186023	13260798	Y	N	N	N	N	N	N	*JPH3*
18	13268589	13299788	Y	N	N	N	N	N	N	*KLHDC4*
18	13341371	13370494	Y	N	N	N	N	N	N	*SLC7A5*
18	13389828	13412237	Y	N	N	N	N	N	N	*CA5A*
18	13425303	13493366	Y	N	N	N	N	N	N	*BANP*
18	14061951	14077096	Y	N	N	N	N	N	N	*GALNS*
18	14096667	14174040	Y	N	N	N	N	N	N	*CBFA2T3*
18	16551061	16610345	Y	N	N	N	N	N	N	*ABCC12*
18	18041277	18392207	Y	N	N	N	N	N	N	*ZNF423*
18	23861829	23927174	Y	N	N	N	N	N	N	*LPCAT2*
18	22541532	22118201	N	N	N	N	N	N	Y	*FTO*
18	39221305	39300130	Y	N	N	N	N	N	N	*PKD1L3*
18	50154685	50077666	N	N	N	N	N	N	Y	*SPTBN4*
19	42771008	42749145	N	N	N	N	N	N	Y	*TTC25*
20	14941022	14836402	N	N	N	N	N	Y	N	*RGS7BP*
20	71208407	71182950	N	N	N	N	N	N	Y	*SLC6A3*
21	35415163	35655955	N	Y	N	N	N	N	N	*STXBP6*
22	55944219	56217921	N	Y	N	N	N	N	N	*ATG7*
22	56530332	56533155	N	N	N	Y	N	N	N	*TRH*
22	59460406	59549010	N	N	N	N	N	N	N	*IQSEC1*
22	10722545	10652411	N	N	N	N	N	N	Y	*TRANK1*
23	18623680	18299317	N	N	N	N	N	N	Y	*Novel*
24	582652	659851	N	N	N	N	N	N	N	*PARD6G*
24	680619	701258	N	N	N	N	N	N	N	*ADNP2*
24	729251	741978	N	N	N	N	N	N	N	*RBFA*
24	743787	776146	N	N	N	N	N	N	N	*NOVEL*
24	33386429	33419545	Y	N	N	N	N	N	N	*ANKRD29*
26	6344781	6348912	N	N	N	N	N	N	N	*MBL2*

26	5017714	5578654	N	Y	N	Y	N	N	N	*PCDH15*
26	6906081	8343629	Y	Y	N	Y	N	N	N	*PRKG1*
26	20694707	20777487	Y	N	Y	N	N	N	N	*DNMBP*
26	20206332	20276715	Y	N	N	N	N	N	N	*CNNM1*
26	20476402	20466104	N	N	N	N	N	N	Y	*SLC25A28*
26	20494069	20524328	Y	N	N	N	N	N	N	*ENTPD7*
26	20613538	20684065	Y	N	N	N	N	N	N	*ABCC2*
26	21670831	21630816	N	N	N	N	N	N	Y	*SLF2*
26	21010622	21035035	Y	N	N	N	N	N	N	*CWF19L1*
27	28963365	28969954	N	N	N	N	N	N	N	*DUSP26*
28	25073149	25191276	N	N	N	N	N	N	Y	*TET1*
28	28777941	28562368	N	N	N	N	N	N	Y	*MICU1*
28	44545580	44597645	N	N	N	N	N	N	N	*ZFAND4*
28	44545580	44597645	N	N	N	N	N	N	N	*NOVEL*
28	44612704	44722610	N	N	N	N	N	N	N	*NOVEL*

- Y indicate the test detect the gene region, N indicate the test did not detect the gene region.
- Red colour lines refer to immunity genes.
- Rsb for Rustaqi X Holstein, revealed 12 genes in Rustaqi (**green colour**): no genes shared between analysis of Rustaqi here & previous genes that appeared in just Iraqi analysis.
- In Rsb Rustaqi X Nellore analysis. 5 genes appeared (**violet colour**), no sharing genes with our previous Iraqi analysis.
- In Rsb Rustaqi X N'Dama G., 26 genes appeared (**brown colour**), 1 gene sharing with previous Iraqi breeds analysis (CD96).
