# Supplementary material for: Genome Analysis Reveals Genetic Admixture and Signature of Selection for Productivity and Environmental Traits in Iraqi Cattle
Source: Front Genet. 2019 Jul 16;10:609. doi: 10.3389/fgene.2019.00609 (PMC6646475; doi:10.3389/fgene.2019.00609)
Supplement: Supplementary file 10 [file Table_10.pdf]

Supplementary Table S10, a: Panther Tool Ontology for Jenoubi breed

Ontology = Biological Process - (*iHS* genes)

Ontology - Category	Gene ID	Gene Name	Panther Family/Subfamily	PANTHER Protein Class
**Biological Regulation** (GO:0065007)	*TNFAIP8*	Tumor necrosis factor alpha-induced protein 8	Tumor necrosis factor alpha-induced protein 8 (PTHR12757:SF3)	-
	SLC4A4	Electrogenic sodium bicarbonate cotransporter 1	Electrogenic sodium bicarbonate cotransporter 1 (PTHR11453:SF10)	Cation transporter
**Cellular Component Organization or Biogenesis** (GO:0071840)	*ATG7*	Uncharacterized protein	Ubiquitin-like modifier-activating enzyme ATG7 (PTHR10953:SF3)	ligase<br>transfer/carrier protein
	SGCD	Uncharacterized protein	Delta-sarcoglycan (PTHR12939:SF6)	cytoskeletal protein
**Cellular Process** (GO:0009987)	*STXBP6*	Syntaxin-binding protein 6 STXBP6 ortholog	Exocyst complex component 1-related (PTHR16092:SF28)	transfer/carrier protein
	MTHFD2L	Uncharacterized protein	Bifunctional methylenetetrahydrofolate dehydrogenase/cyclohydrolase 2-related (PTHR10025:SF42)	dehydrogenase<br>hydrolase
	ATG7	Uncharacterized protein	Ubiquitin-like modifier-activating enzyme ATG7 (PTHR10953:SF3)	ligase<br>transfer/carrier protein
	PRKG1	cGMP-dependent protein kinase 1	SUBFAMILY NOT NAMED (PTHR24353:SF68)	-

	TNFAIP8	Tumor necrosis factor alpha-induced protein 8	Tumor necrosis factor alpha-induced protein 8 (PTHR12757:SF3)	-
	SLC4A4	Electrogenic sodium bicarbonate cotransporter 1	Electrogenic sodium bicarbonate cotransporter 1 (PTHR11453:SF10)	cation transporter
	SGCD	Uncharacterized protein	Delta-sarcoglycan (PTHR12939:SF6)	cytoskeletal protein
**Developmental Process** (GO:0032502)	*EPHA5*	Uncharacterized protein	Ephrin type-A receptor 5 (PTHR24416:SF17)	-
	PCDH15	Uncharacterized protein	Protocadherin-15 (PTHR24028:SF11)	-
	TNFAIP8	Tumor necrosis factor alpha-induced protein 8	Tumor necrosis factor alpha-induced protein 8 (PTHR12757:SF3)	-
	SGCD	Uncharacterized protein	Delta-sarcoglycan (PTHR12939:SF6)	cytoskeletal protein
**Localization** (GO:0051179)	*STXBP6*	Syntaxin-binding protein 6	Exocyst complex component 1-related (PTHR16092:SF28)	transfer/carrier protein
	SLC4A4	Electrogenic sodium bicarbonate cotransporter 1	Electrogenic sodium bicarbonate cotransporter 1 (PTHR11453:SF10)	cation transporter
**Metabolic Process** (GO:0008152)	*ATG7*	Uncharacterized protein	Ubiquitin-like modifier-activating enzyme ATG7 (PTHR10953:SF3)	ligase<br>transfer/carrier protein
	PRKG1	cGMP-dependent protein kinase 1	SUBFAMILY NOT NAMED (PTHR24353:SF68)	-

**Multicellular Organismal Process** (GO:0032501)				
	SGCD	Uncharacterized protein	Delta-sarcoglycan (PTHR12939:SF6)	cytoskeletal protein
**Response to Stimulus** (GO:0050896)	*ATG7*	Uncharacterized protein	Ubiquitin-like modifier-activating enzyme ATG7 (PTHR10953:SF3)	ligase<br>transfer/carrier protein

Supplementary Table S10, b: Panther Tool Ontology for Jenoubi breed

Ontology = Molecular Function - (Jenoubi breed – *iHS* genes – HD data)

Ontology - Category	Gene ID	Gene Name	Panther Family/Subfamily	PANTHER Protein Class
**Binding** (GO:0005488)	*STXBP6*	Syntaxin-binding protein 6	Exocyst complex component 1-related (PTHR16092:SF28)	Transfer/carrier protein
**Catalytic Activity** (GO:0003824)	*MTHFD2L*	Uncharacterized protein	Bifunctional methylenetetrahydrofolate dehydrogenase/cyclohydrolase 2-related (PTHR10025:SF42)	Dehydrogenase hydrolase
	ATG7	Uncharacterized protein	Ubiquitin-like modifier-activating enzyme ATG7 (PTHR10953:SF3)	Ligase transfer/carrier protein
	TNFAIP8	Tumor necrosis factor alpha-induced protein 8	Tumor necrosis factor alpha-induced protein 8 (PTHR12757:SF3)	-
**Transporter Activity** (GO:0005215)	*SLC4A4*	Electrogenic sodium bicarbonate cotransporter 1 SLC4A4 ortholog	Electrogenic sodium bicarbonate cotransporter 1 (PTHR11453:SF10)	Cation transporter

Supplementary Table S10, c: Panther Tool Ontology for Jenoubi breed

Ontology = Protein Class - (Jenoubi breed – *iHS* genes – HD data)

Ontology - Category	Gene ID	Gene Name	Panther Family/Subfamily	PANTHER Protein Class
**Cytoskeletal protein** (PC00085)	*SGCD*	Uncharacterized protein	Delta-sarcoglycan (PTHR12939:SF6)	Cytoskeletal protein
**Hydrolase** (PC00121)	*MTHFD2L*	Uncharacterized protein	Bifunctional methylenetetrahydrofolate dehydrogenase/cyclohydrolase 2-related (PTHR10025:SF42)	Dehydrogenase hydrolase
**Ligase (PC00142)**	*ATG7*	Uncharacterized protein	Ubiquitin-like modifier-activating enzyme ATG7 (PTHR10953:SF3)	Ligase transfer/carrier protein
**Oxidoreductase (PC00176)**	*MTHFD2L*	Uncharacterized protein	Bifunctional methylenetetrahydrofolate dehydrogenase/cyclohydrolase 2-related (PTHR10025:SF42)	Dehydrogenase hydrolase
**Transfer/carrier protein (PC00219)**	*STXBP6*	Syntaxin-binding protein 6	Exocyst complex component 1-related (PTHR16092:SF28)	Transfer/carrier protein
	ATG7	Uncharacterized protein	Ubiquitin-like modifier-activating enzyme ATG7 (PTHR10953:SF3)	Ligase transfer/carrier protein
**Transporter (PC00227)**	*SLC4A4*	Electrogenic sodium bicarbonate cotransporter 1	Electrogenic sodium bicarbonate cotransporter 1 (PTHR11453:SF10)	Cation transporter

Supplementary Table S10, d: Panther Tool Ontology for Jenoubi breed

Ontology = Cellular Components (Jenoubi breed –*iHS* genes - HD data)

**Ontology - Category**	**Gene ID**	**Gene Name**	**Panther Family/Subfamily**	**PANTHER Protein Class**
**Cell Part** (GO:0044464)	*STXBP6*	Syntaxin-binding protein 6	Exocyst complex component 1-related (PTHR16092:SF28)	Transfer/carrier protein
	MTHFD2L	Uncharacterized protein	Bifunctional methylenetetrahydrofolate dehydrogenase/cyclohydrolase 2-related (PTHR10025:SF42)	Dehydrogenase hydrolase
	ATG7	Uncharacterized protein	Ubiquitin-like modifier-activating enzyme ATG7 (PTHR10953:SF3)	Ligase transfer/carrier protein
	TNFAIP8	Tumor necrosis factor alpha-induced protein 8	Tumor necrosis factor alpha-induced protein 8 (PTHR12757:SF3)	-
	SLC4A4	Electrogenic sodium bicarbonate cotransporter 1	Electrogenic sodium bicarbonate cotransporter 1 (PTHR11453:SF10)	Cation transporter
	SGCD	Uncharacterized protein	Delta-sarcoglycan (PTHR12939:SF6)	Cytoskeletal protein
**Macromolecular Complex** (GO:0032991)	*STXBP6*	Syntaxin-binding protein 6	Exocyst complex component 1-related (PTHR16092:SF28)	Transfer/carrier protein
	SGCD	Uncharacterized protein	Delta-sarcoglycan (PTHR12939:SF6)	Cytoskeletal protein

**Membrane** (GO:0016020)	*SLC4A4*	Electrogenic sodium bicarbonate cotransporter 1	Electrogenic sodium bicarbonate cotransporter 1 (PTHR11453:SF10)	Cation transporter
**Organelle** (GO:0043226)	*MTHFD2L*	Uncharacterized protein	bifunctional methylenetetrahydrofolate dehydrogenase/cyclohydrolase 2-related (PTHR10025:SF42)	Dehydrogenase hydrolase
