# Supplementary material for: Genome Analysis Reveals Genetic Admixture and Signature of Selection for Productivity and Environmental Traits in Iraqi Cattle
Source: Front Genet. 2019 Jul 16;10:609. doi: 10.3389/fgene.2019.00609 (PMC6646475; doi:10.3389/fgene.2019.00609)
Supplement: Supplementary file 11 [file Table_11.pdf]

Supplementary Table S11, a: Enrichr Tool Ontology for Jenoubi *iHS*

(Biological Process)

Index	Name	P-value	Adjusted p-value	Z-score	Combined score
1	positive regulation of apoptotic process (GO:0043065)	0.009527	0.02848	-3.76	13.38
2	sensory perception of light stimulus (GO:0050953)	0.005836	0.02413	-3.34	12.45
3	regulation of GTPase activity (GO:0043087)	0.0004508	0.01984	-2.86	11.21
4	hippocampus development (GO:0021766)	0.007129	0.02413	-2.99	11.12
5	regulation of insulin secretion involved in cellular response to glucose stimulus (GO:0061178)	0.006482	0.02413	-2.75	10.26
6	cardiac muscle tissue development (GO:0048738)	0.007129	0.02413	-2.60	9.70
7	heart contraction (GO:0060047)	0.006482	0.02413	-2.52	9.39
8	positive regulation of CREB transcription factor activity (GO:0032793)	0.009709	0.02848	-2.58	9.19
9	negative regulation of platelet aggregation (GO:0090331)	0.004542	0.02413	-2.39	8.89

10	equilibrioception (GO:0050957)	0.003894	0.02413	-2.22	8.26

Supplementary Table S11, b: Show Enrichr Tool Ontology for Jenoubi *iHS*

(Molecular Function)

Index	Name	P-value	Adjusted p-value	Z-score	Combined score
1	cysteine-type endopeptidase inhibitor activity involved in apoptotic process (GO:0043027)	0.01549	0.02711	-3.26	11.76
2	transmembrane-ephrin receptor activity (GO:0005005)	0.005189	0.01816	-2.90	11.63
3	calcium channel regulator activity (GO:0005246)	0.009709	0.02265	-2.91	11.02
4	actin binding (GO:0003779)	0.07829	0.1096	-4.35	9.62
5	GPI-linked ephrin receptor activity (GO:0005004)	0.003246	0.01816	-2.16	8.65
6	protein serine/threonine kinase activity (GO:0004674)	0.1763	0.2056	-4.92	7.78
7	protein homodimerization activity (GO:0042803)	0.3198	0.3198	-6.54	7.46
