# Supplementary figures and images for: Genome Analysis Reveals Genetic Admixture and Signature of Selection for Productivity and Environmental Traits in Iraqi Cattle
Source: Front Genet. 2019 Jul 16;10:609. doi: 10.3389/fgene.2019.00609 (PMC6646475; doi:10.3389/fgene.2019.00609)

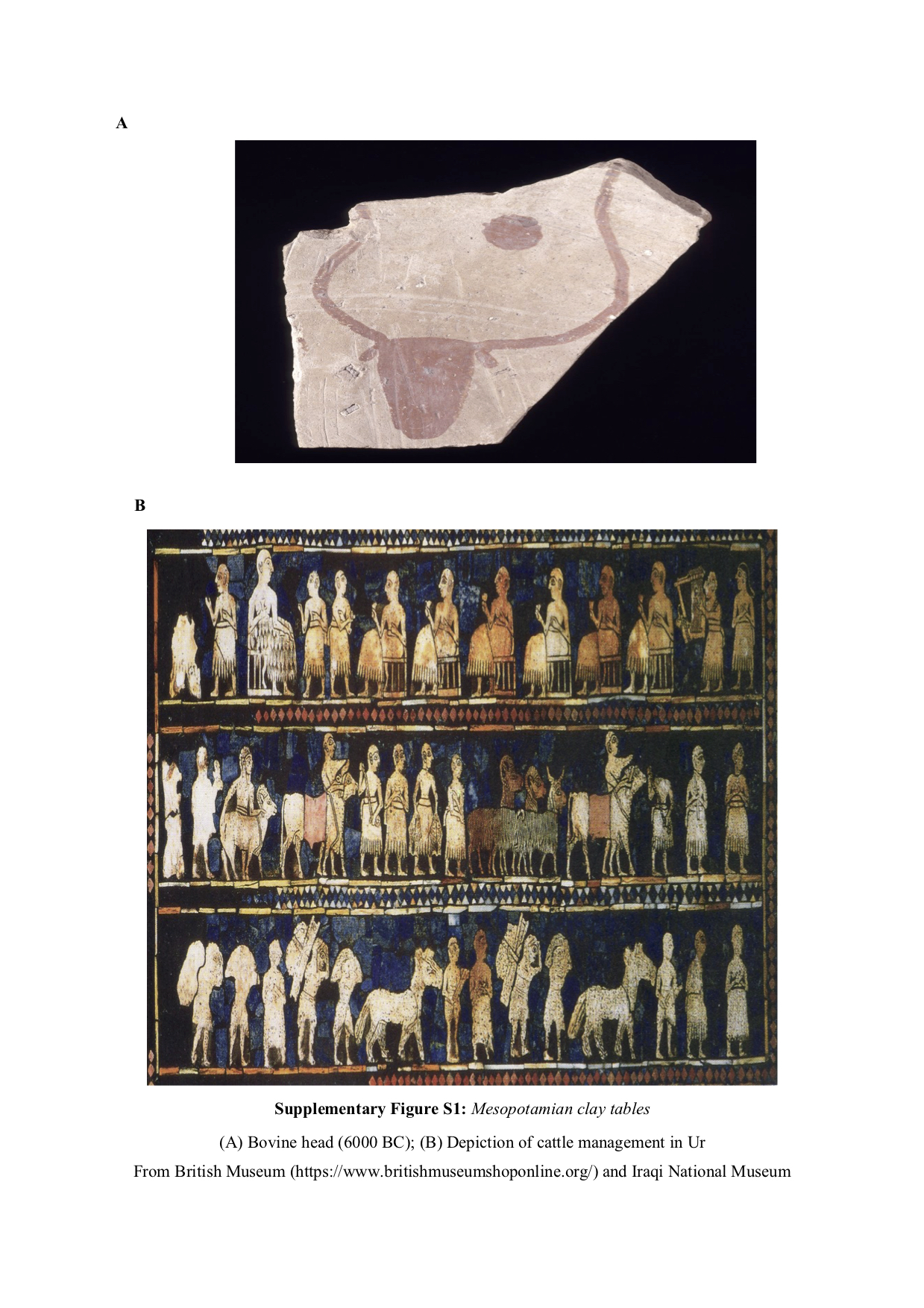

Supplement: Supplementary file 13 [file Image_1.jpeg]

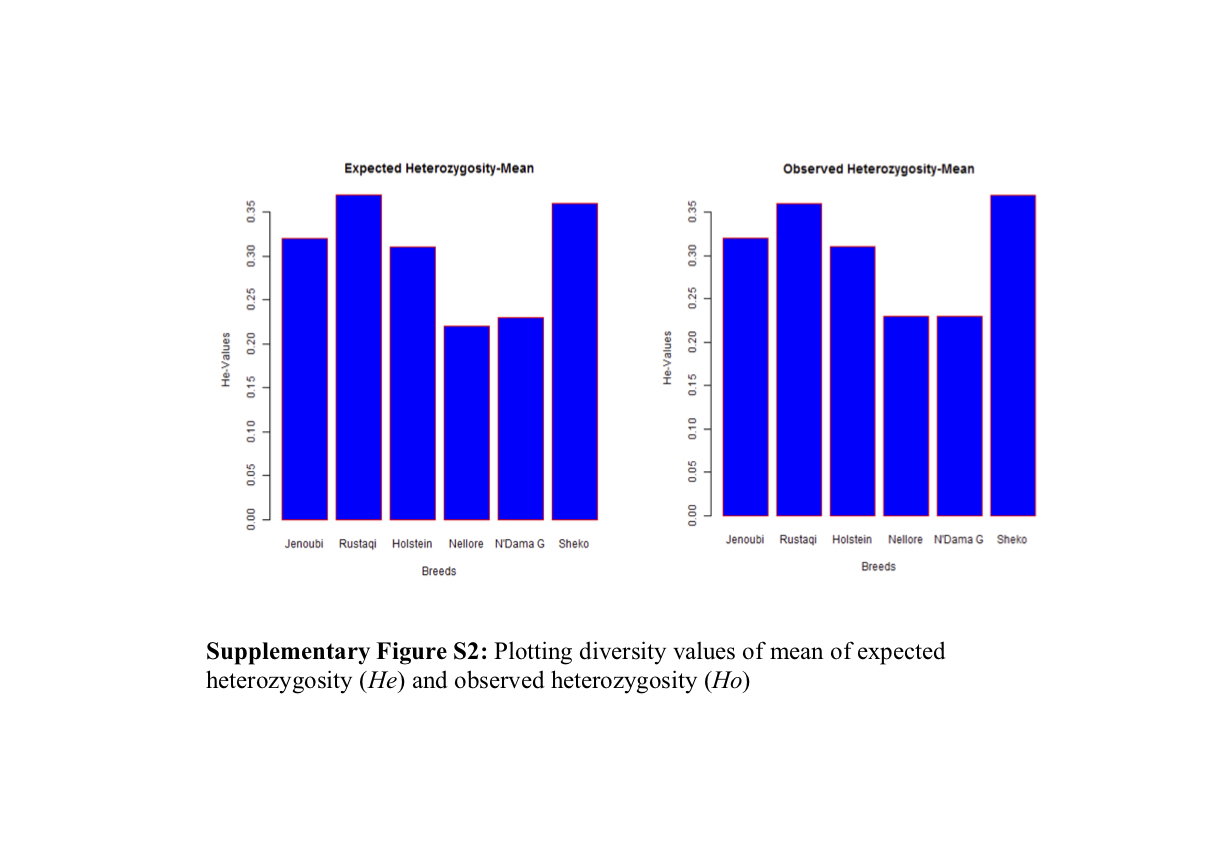

Supplement: Supplementary file 14 [file Image_2.jpeg]

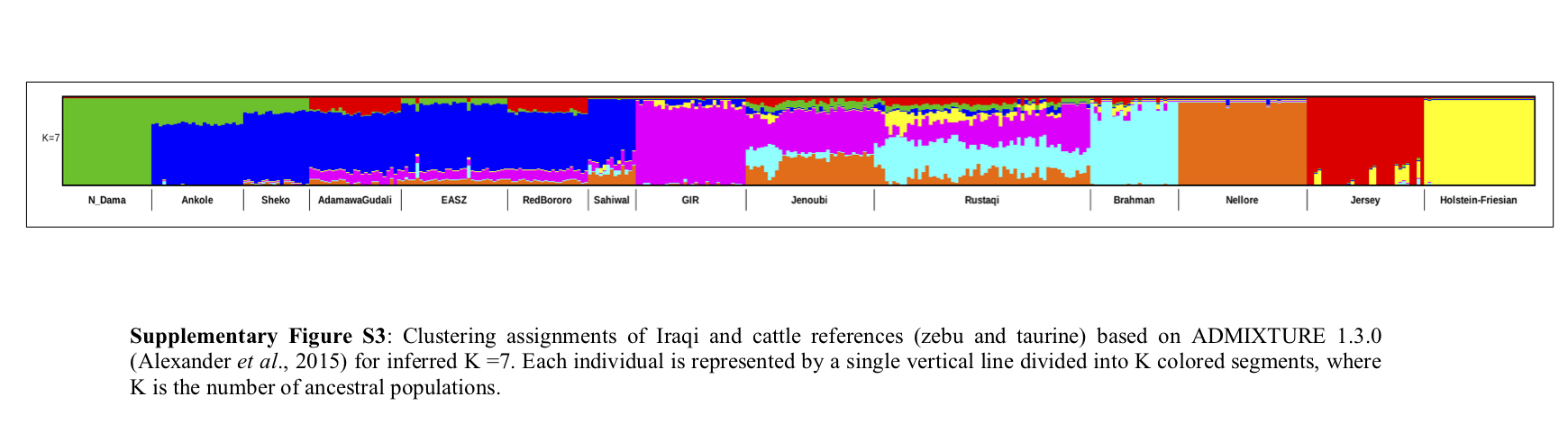

Supplement: Supplementary file 15 [file Image_3.jpeg]

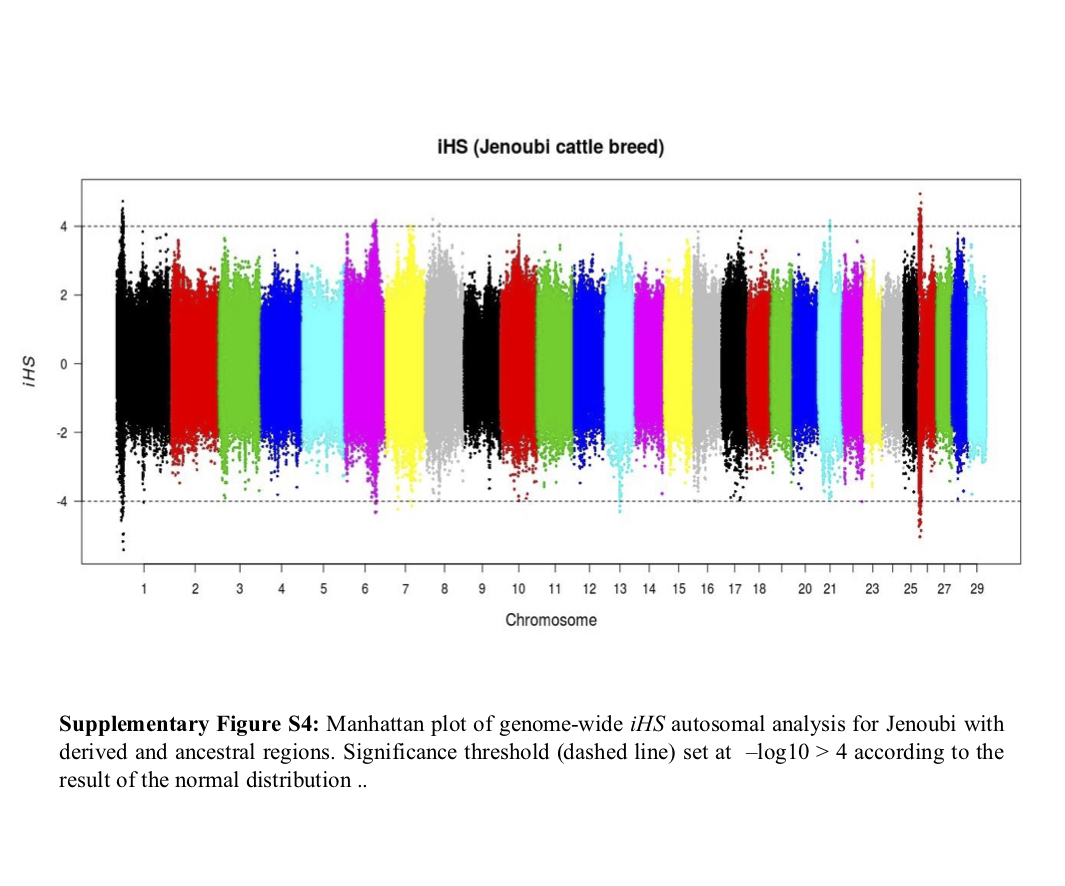

Supplement: Supplementary file 16 [file Image_4.jpeg]

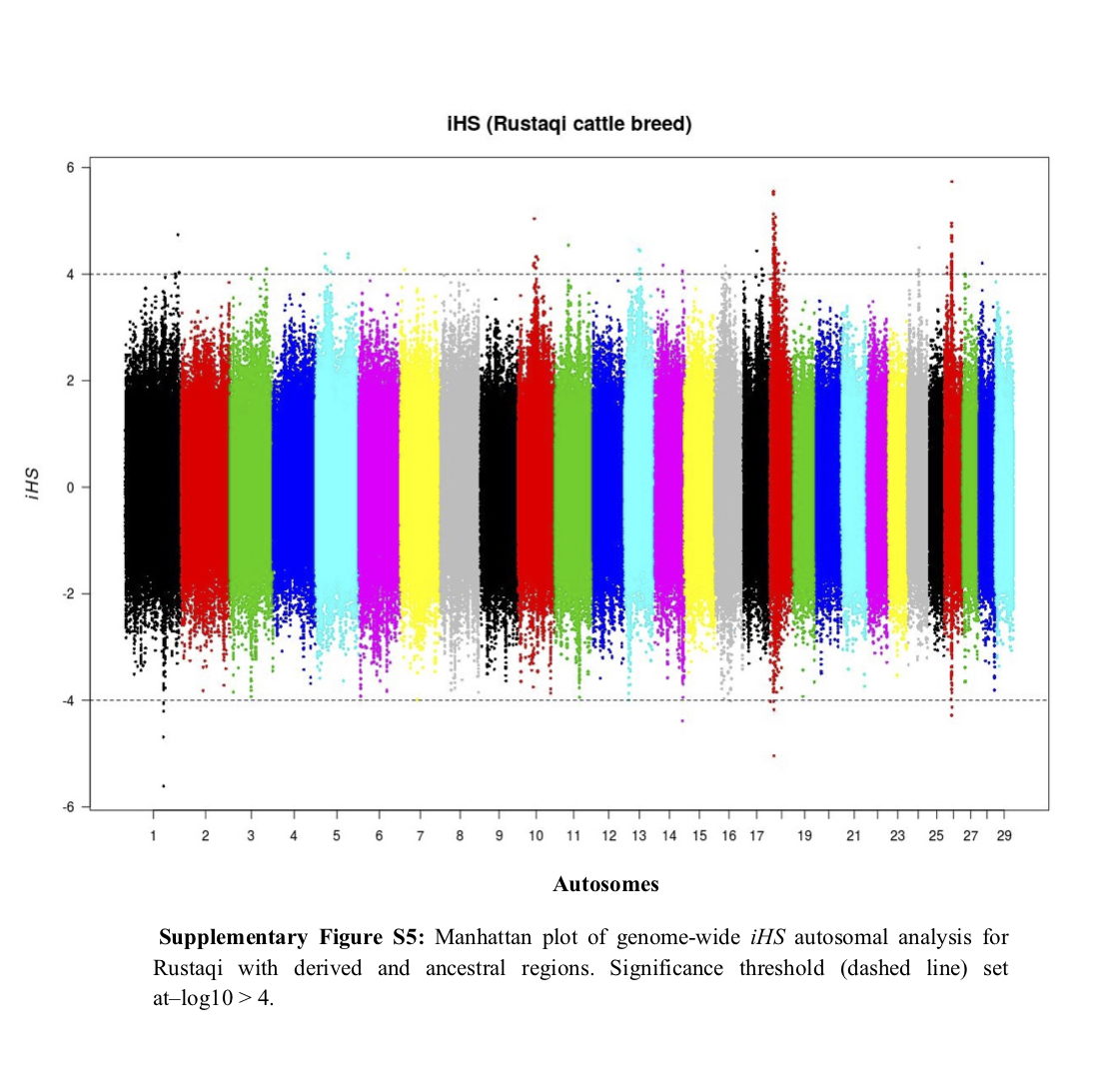

Supplement: Supplementary file 17 [file Image_5.jpeg]

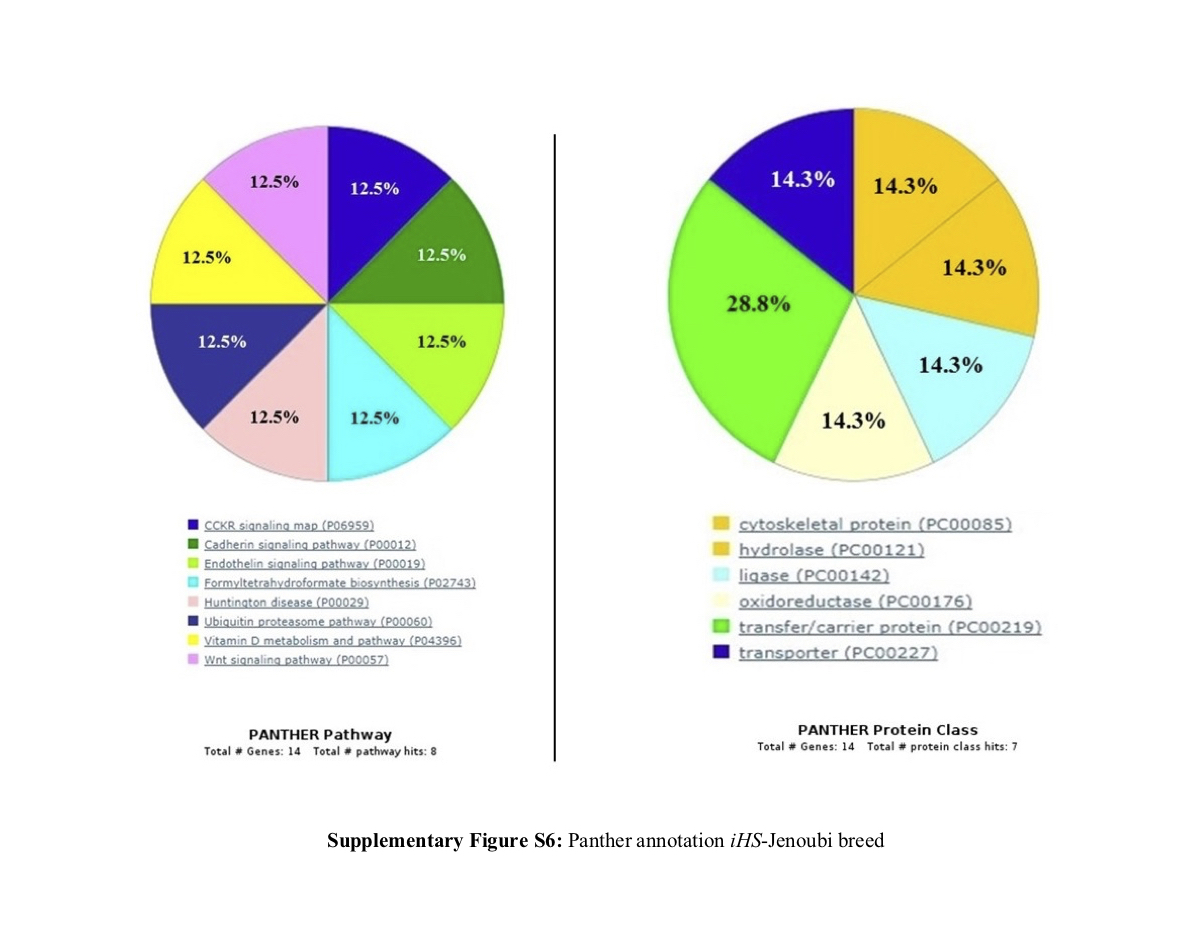

Supplement: Supplementary file 18 [file Image_6.jpeg]

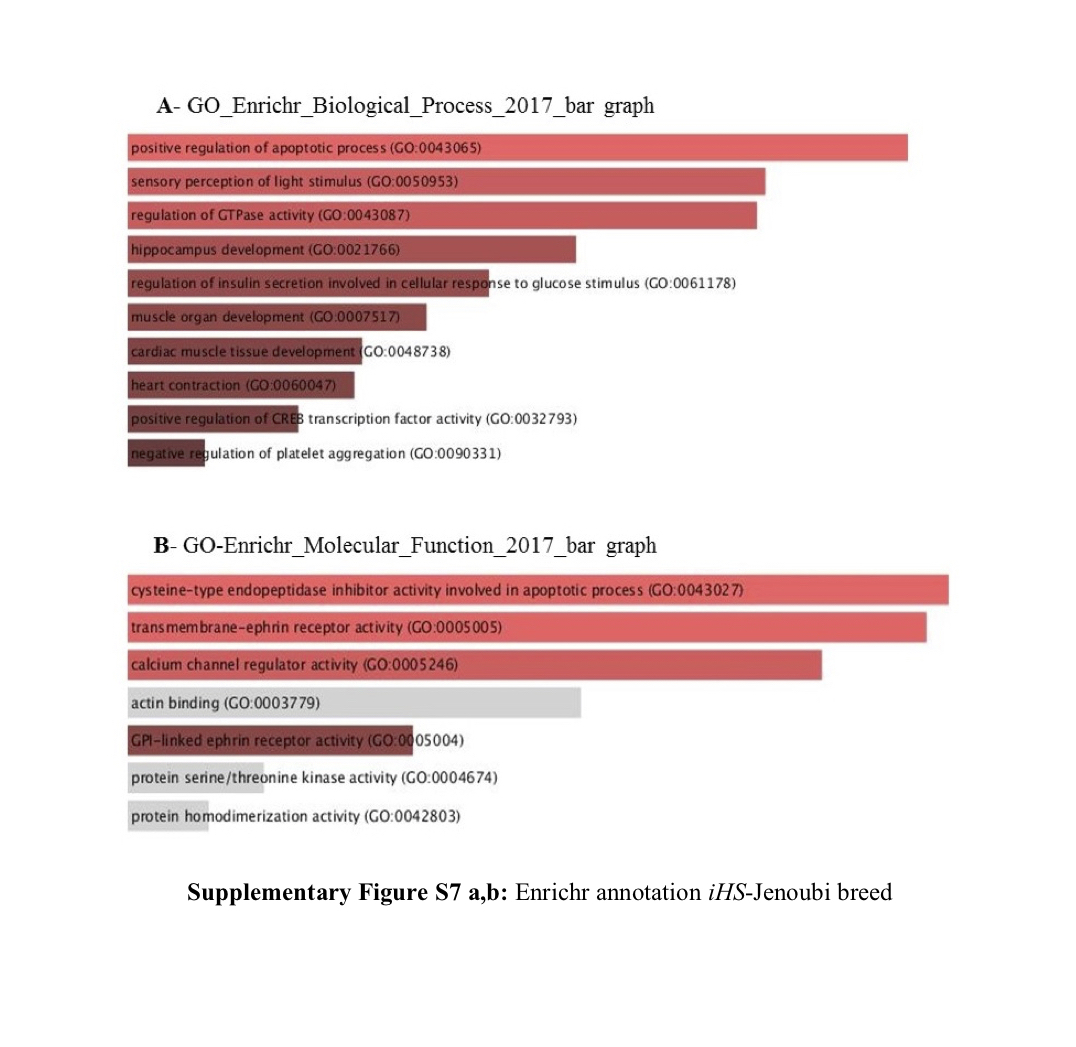

Supplement: Supplementary file 19 [file Image_7.jpeg]

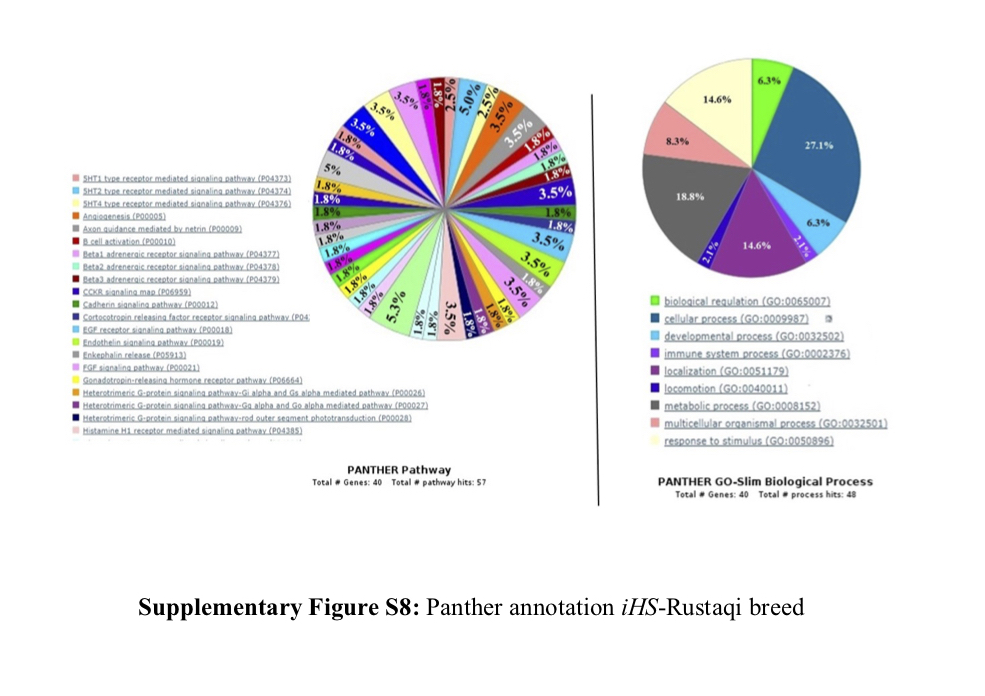

Supplement: Supplementary file 20 [file Image_8.jpeg]

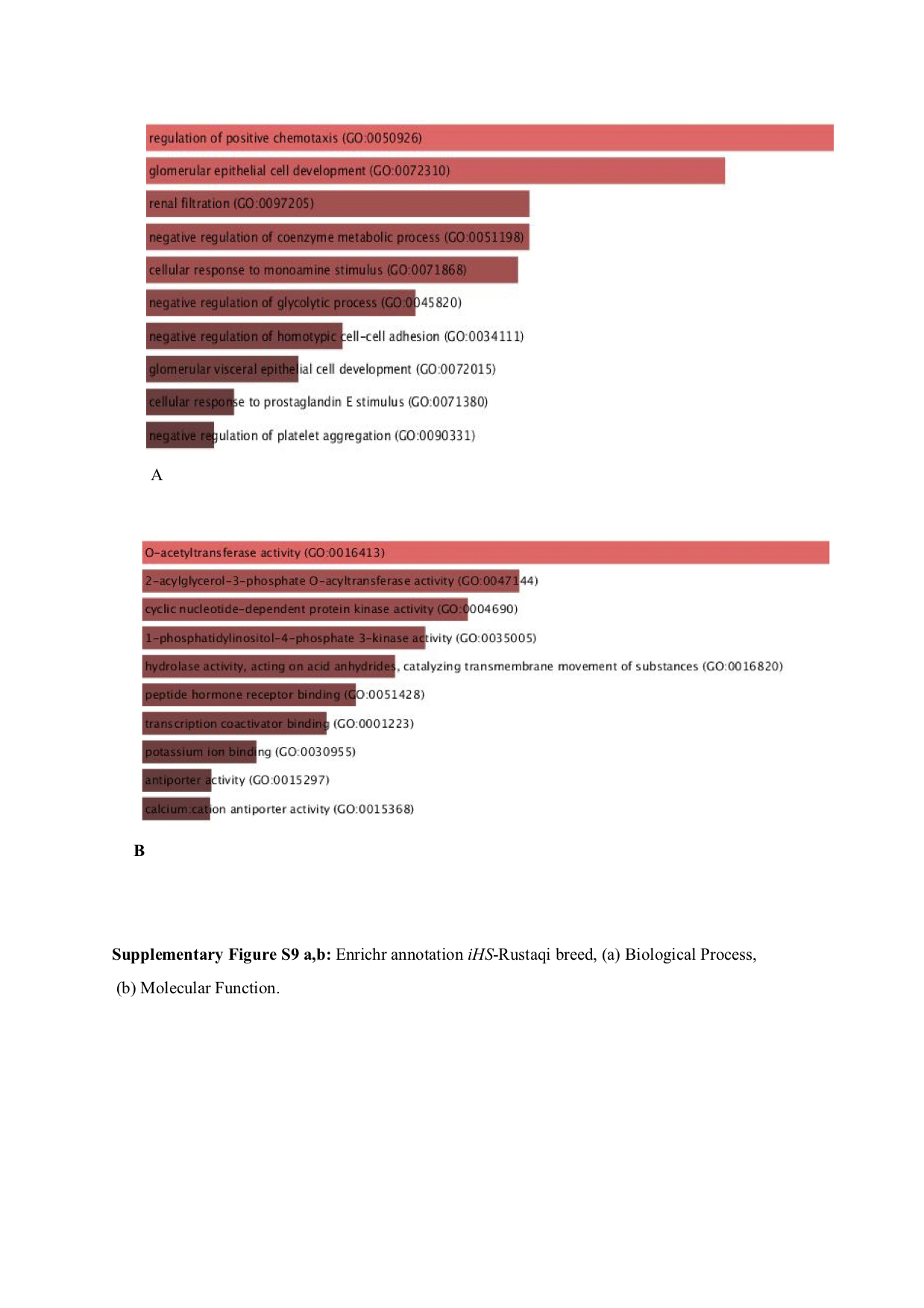

Supplement: Supplementary file 21 [file Image_9.jpeg]

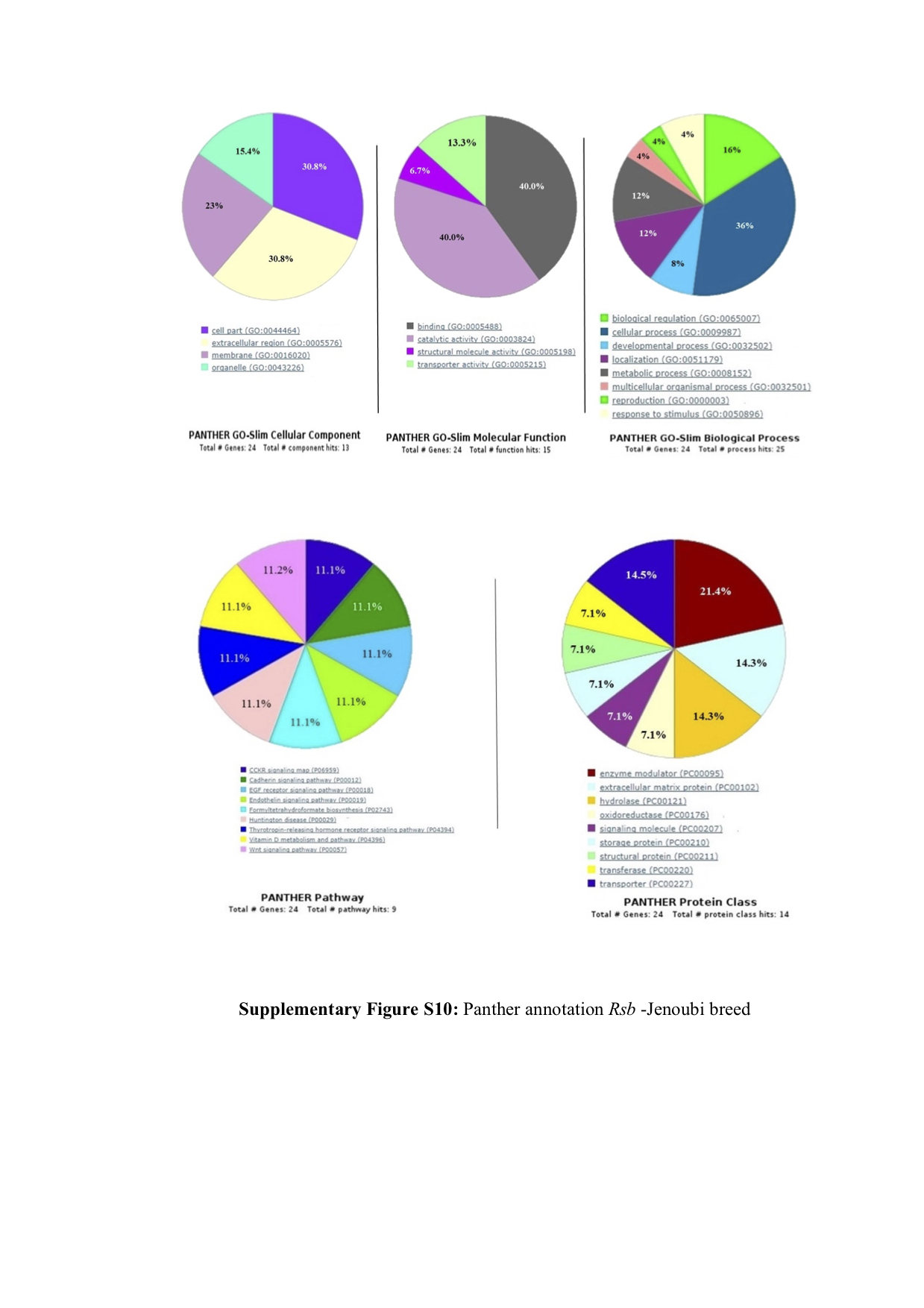

Supplement: Supplementary file 22 [file Image_10.jpeg]

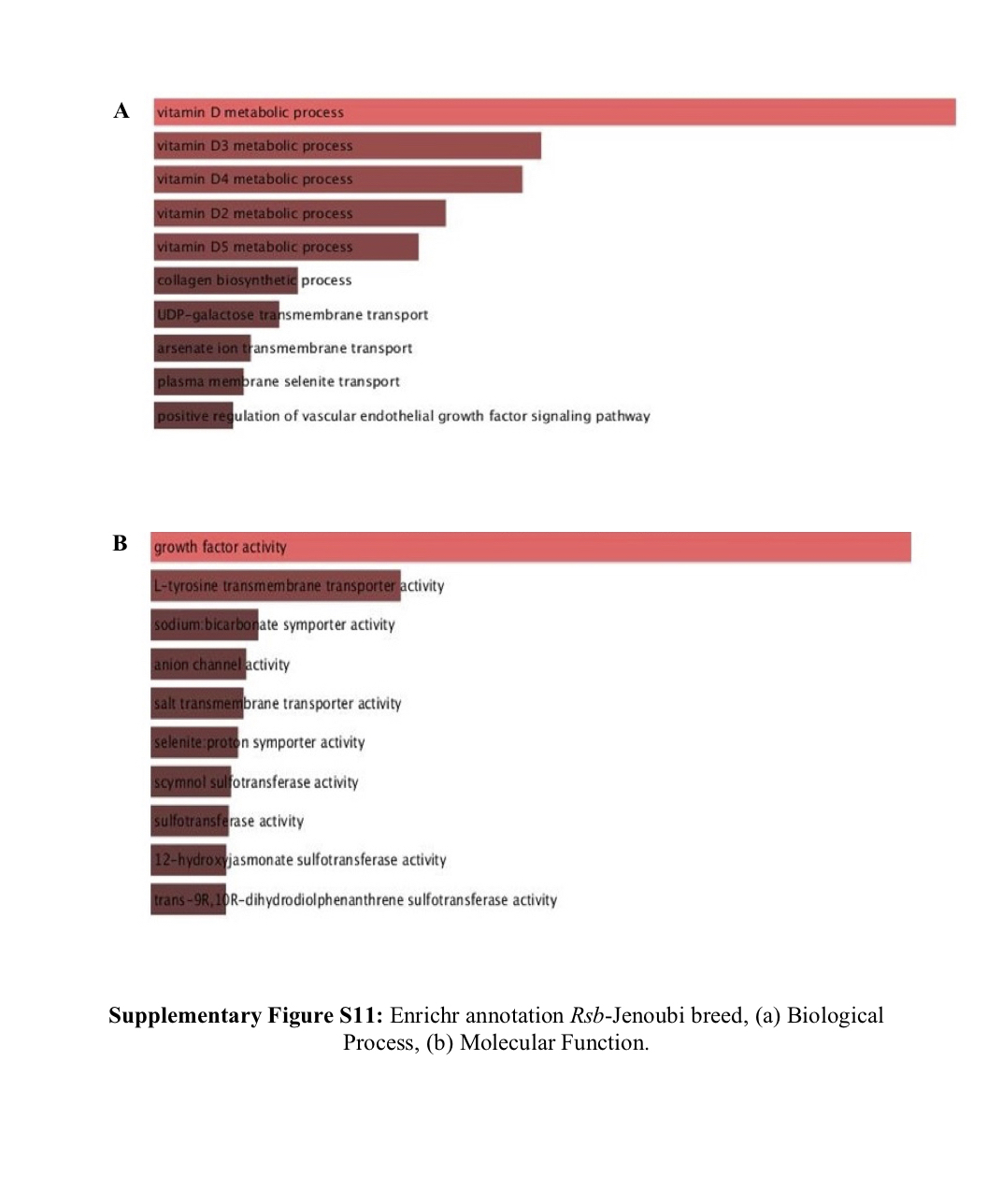

Supplement: Supplementary file 23 [file Image_11.jpeg]
